# Effect of Tigecycline on the Homeostasis of Human Epidermal Melanocytes and Fibroblasts

**DOI:** 10.3390/ijms26188939

**Published:** 2025-09-13

**Authors:** Zuzanna Rzepka, Marta Karkoszka-Stanowska, Krzysztof Marciniec, Magdalena Zdybel, Barbara Pilawa, Dorota Wrześniok

**Affiliations:** 1 Department of Pharmaceutical Chemistry, Faculty of Pharmaceutical Sciences in Sosnowiec, Medical University of Silesia in Katowice, Jagiellońska 4, 41-200 Sosnowiec, Poland; zrzepka@sum.edu.pl (Z.R.); marta.karkoszka@sum.edu.pl (M.K.-S.); 2 Department of Organic Chemistry, Faculty of Pharmaceutical Sciences in Sosnowiec, Medical University of Silesia in Katowice, Jagiellońska 4, 41-200 Sosnowiec, Poland; kmarciniec@sum.edu.pl; 3 Department of Biophysics, Faculty of Pharmaceutical Sciences in Sosnowiec, Medical University of Silesia in Katowice, Jedności 8, 41-200 Sosnowiec, Poland; mzdybel@sum.edu.pl (M.Z.); bpilawa@sum.edu.pl (B.P.)

**Keywords:** tigecycline, melanin, melanocytes, fibroblasts, skin cells

## Abstract

Tigecycline is an antibiotic belonging to the glycylcycline group of tetracyclines. Similar to other tetracycline derivatives, tigecycline is used in dermatology because of its bacteriostatic effect. Despite an overall favorable safety profile, tetracyclines are associated with a spectrum of cutaneous adverse effects, notably pigmentary disorders and phototoxic reactions. These dermatologic manifestations are presumed to result from tigecycline’s affinity for melanin biopolymer and its subsequent accumulation within the pigment-containing tissues. This study aimed to assess the impact of tigecycline on human normal skin cell homeostasis varied by melanin content. The study was conducted on HEMn-LP melanocytes and human dermal fibroblasts. The aim was achieved by determining the cell number, cell cycle, mitochondrial potential, and redox homeostasis and determining in silico the possibility of binding tigecycline to melanin biopolymers. In this study, it was shown that the cells more sensitive to tigecycline were HEMn-LP melanocytes. The obtained results showed that tigecycline decreased cell number in a dose-dependent manner. In addition, tigecycline was shown to reduce mitochondrial potential, increase the level of oxidized thiols, and increase ROS content in melanocytes, contributing to oxidative stress. In silico studies have shown that the binding of tigecycline to melanin may play a role in the induction of the toxic effects of tigecycline on the skin.

## 1. Introduction

Tetracycline antibiotics, due to their pharmacological properties, high safety and efficacy profile, as well as broad spectrum of action, have been of interest to researchers for years. The main indications for tetracycline therapy include bacterial infections of the skin and soft tissues (acne, rosacea), infections of the respiratory tract, genitourinary tract (chalmydiosis, syphilis, gonorrhoea), and zoonoses (Lyme disease, brucellosis, or anthrax) [1]. It is worth noting that, in addition to their bacteriostatic activity, tetracyclines exhibit a number of non-antibiotic properties, e.g., immunomodulation, that allow them to be used in the treatment of scleroderma, periodontitis, tumors, or rheumatoid arthritis [2,3,4].

Tigecycline belongs to the group of tetracycline antibiotics, and is a first member of a unique glycylcycline group referred to as one of the generations of tetracyclines [5,6]. The main structural difference that distinguishes tigecycline from other tetracyclines is the presence of a t-butylglycylamide moiety at the C9 position of the A ring [7]. The bacteriostatic effect of tigecycline is due to its ability to bind to the 30S subunit of the bacterial ribosome, preventing aminoacyl-tRNA from attaching to the ribosomal complex, leading to inhibition of protein synthesis in bacterial cells. The presence of the glycyl group of tigecycline overcomes established bacterial resistance mechanisms, which include efflux pumps, tetracycline-degrading enzymes, or the protective ribosome proteins Tet (M) and Tet (O). The above facts make tigecycline a suitable drug for the treatment of bacterial infections caused by microorganisms resistant to other antibiotics, including multi-resistant strains of Gram-positive and Gram-negative bacteria [7,8].

Despite the good safety profile, tetracyclines, including tigecycline, can cause a variety of cutaneous adverse effects, which include pigmentary disturbances and phototoxic reactions [9]. It is likely that the aforementioned side effects may be related to the binding of tigecycline to melanin biopolymers and accumulation of the drug in the skin. Melanin is a naturally occurring pigment in cells called melanocytes formed by a multi-step tyrosine oxidation process, whose main purpose, due to its intracellular location, is to protect the cell’s genetic material from the damaging effects of UV radiation [10]. There are two types of melanin, eumelanin and pheomelanin, which are synthesized in mammalian melanocytes. A common feature of both types of melanin is the absorption of radiation from the UV range. Eumelanin is a black-brown polymer composed of two subunits—5,6-dihydroxyindole-2-carboxylic acid (DHICA) and 5,6-dihydroxyindole (DHI)—varied by oxidation degree [11,12]. Pheomelanin takes on a yellow to reddish-brown color and contains sulfur and nitrogen atoms in its structure. Differences in chemical structure and physicochemical properties mean that eumelanin plays the role of a photoprotective substance in the cell, which has natural oxydo-reductive properties due to superoxide dismutase-like activity, while pheomelanin plays the role of a photosensitizing substance due to the presence of 1,4-benzothiazine and benzothiazole moieties [13,14,15,16,17]. Nevertheless, the study by Szewczyk et al. [18] shows that synthetic DHICA-melanin generates singlet oxygen most efficiently under short-wavelength visible light and also quenches it rapidly, indicating a key role of singlet oxygen in its aerobic photochemistry. This distinct behavior suggests that DHICA units, rather than DHI or DOPA components, are likely the main photoreactive sites in eumelanin under UVA–visible light. In vivo the process of melanin synthesis leads to obtaining a mixture of eumelanin and pheomelanin polymers that determines skin phenotypes classified in the Fitzpatrick scale (I-VI phenotype) [19,20].

Apart from photoprotective role, melanin is a biopolymer with chelating properties, capable of binding various substances, including drugs. Drug interactions with melanin can affect their pharmacokinetics, efficacy, and side effects, especially in rich pigmented tissues, such as skin. On the one hand, the polyanionic nature of melanins makes them capable of forming bonds with xenobiotics, the part of cytoprotective properties of melanocytes, while on the other hand, the formation of drug–melanin complexes results in the formation of a long-term drug reservoir at higher concentrations than observed in plasma, and significantly increases the occurrence of adverse effects [21,22].

The aim of the present study was to analyze the effect of tigecycline on the homeostasis of human normal skin cells varied by the levels of melanin pigments—fibroblasts (HDF) and melanocytes (HEMn-LP). Viability, proliferation, cell cycle progression, mitochondrial potential, and thiol status were tested for both cell types. The results indicated that tigecycline was more harmful towards pigment-containing cells. Therefore, we assessed the ability of tigecycline to form complexes with melanin using an in silico method. In addition, we used EPR to test whether the binding of the drug to melanin affects the overproduction of free radicals in melanocytes. The results could contribute to clarifying whether melanin plays a role in the pathomechanism of adverse effects of tigecycline in pigmented tissues.

## 2. Results

### 2.1. The Impact of Tigecycline on Proliferation and Viability of Human Skin Cells

**Figure 1:**
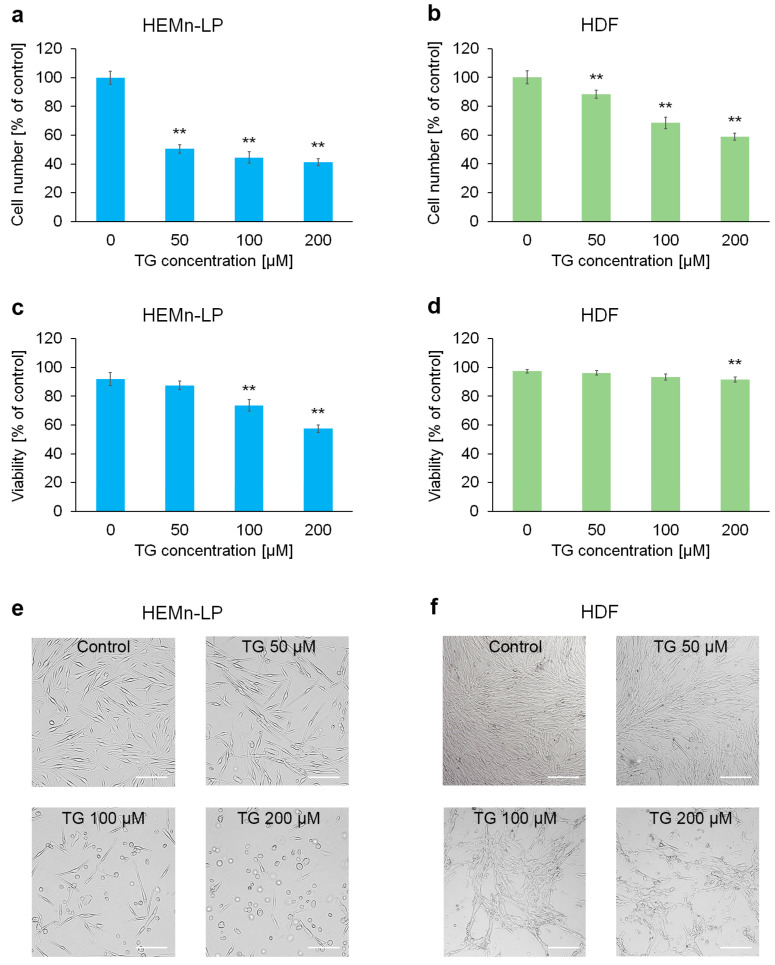
The effect of tigecycline (TG) on proliferation (**a**,**b**), viability (**c**,**d**), and morphology (**e**,**f**) of normal human skin cells: melanocytes (HEMn-LPs) and fibroblasts (HDFs). All analyses were performed after a 48 h incubation of the cells with TG at a concentration of 50, 100, or 200 µM. Bar graphs show the mean value ± SD of three independent experiments; ** *p* < 0.01. In microscopic images, scale bar = 100 µm.

In the first phase of the study, the effect of tigecycline on survival and growth of the skin cells population was assessed by image cytometry. Tests were performed on cell types differing in melanin content—normal human melanocytes (HEMn-LPs) and normal human fibroblasts (HDFs). Prior to analysis, cells were incubated for 48 h with tigecycline solutions at concentrations of 50, 100, and 200 µM. The results from cell count and viability assay are presented in Figure 1a–d. The study showed greater reduction in cell number (Figure 1a) and survival (Figure 1c) compared to controls in the case of HEMn-LPs. Thus, pigmented cells are more sensitive than fibroblasts to the cytotoxic effects of tigecycline. Microscopic observations presented in Figure 1e,f also confirmed this conclusion—melanocytes treated with 100 µM and 200 µM tigecycline had a more altered morphology and features of apoptosis, such as a spherical shape and a tendency to detach.

### 2.2. Effects of Tigecycline on the Cell Cycle of Melanocytes and Fibroblasts

**Figure 2:**
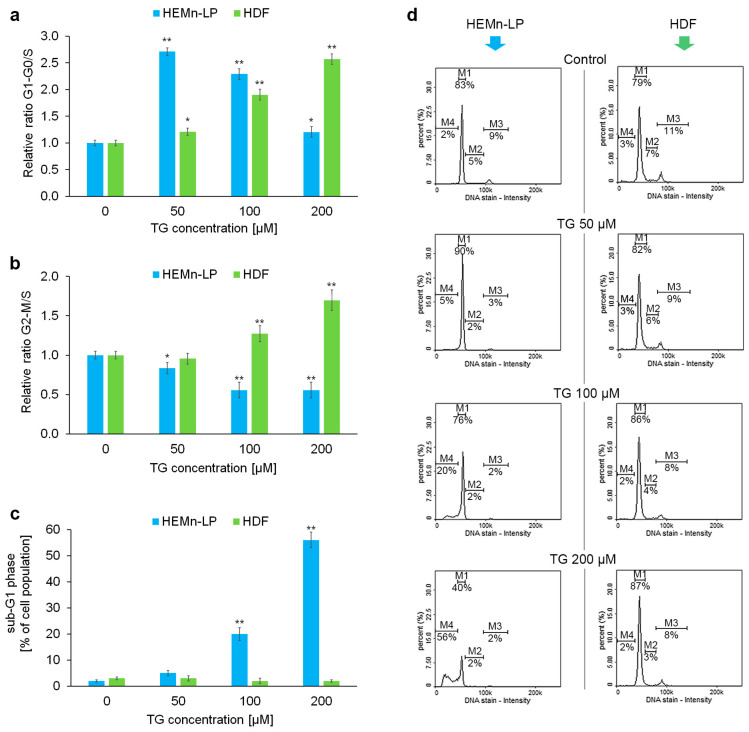
The impact of tigecycline (TG) on the cell cycle distribution of normal human melanocytes (HEMn-LPs) and fibroblasts (HDFs). The cells were cultured for 48 h with TG at a concentration of 50, 100, and 200 μM. Relative G1/G0-to-S ratio (**a**) and G2/M-to-S ratio (**b**) were calculated (the value for the untreated control was set as 1). The percentage of cells in sub-G1 phase is shown (**c**). Bars represent the mean ± SD (standard deviation) of 3 independent experiments, * *p* < 0.05, ** *p* < 0.01 vs. control. Representative histograms from the analysis (**d**) show the populations of cells in the sub-G1 (M1), G1/G0 (M2), S (M3), and G2/M (M4) phases.

The next stage of our in vitro experimental panel was cytometric cell cycle analysis. The results are presented in Figure 2. Based on the percentage of cells in each phase of the cell cycle, the G1/G0 to S ratio and the G2/M to S ratio were calculated to determine whether cell cycle arrest reached significance at the G1 or G2/M checkpoint. Melanocytes treated with tigecycline had a disrupted transition from the G1/G0 phase (Figure 2a,b). This effect was particularly noticeable for tigecycline concentrations of 50 µM. The drug at high concentrations (100 µM, 200 µM) caused a significant increase in the percentage of melanocytes in the sub-G1 phase (Figure 2c), indicating DNA fragmentation, which may suggest apoptosis. Cell cycle analysis of fibroblasts treated with tigecycline showed different changes than for pigmented cells. As shown in the bar graphs in Figure 2a,b, the drug induced a concentration-dependent growth arrest in the G1 and G2/M phase. Moreover, no increase in the percentage of fibroblasts with fragmented DNA, i.e., in the sub-G1 phase, was observed.

### 2.3. Mitochondrial Potential in Melanocytes and Fibroblasts Treated with Tigecycline

**Figure 3:**
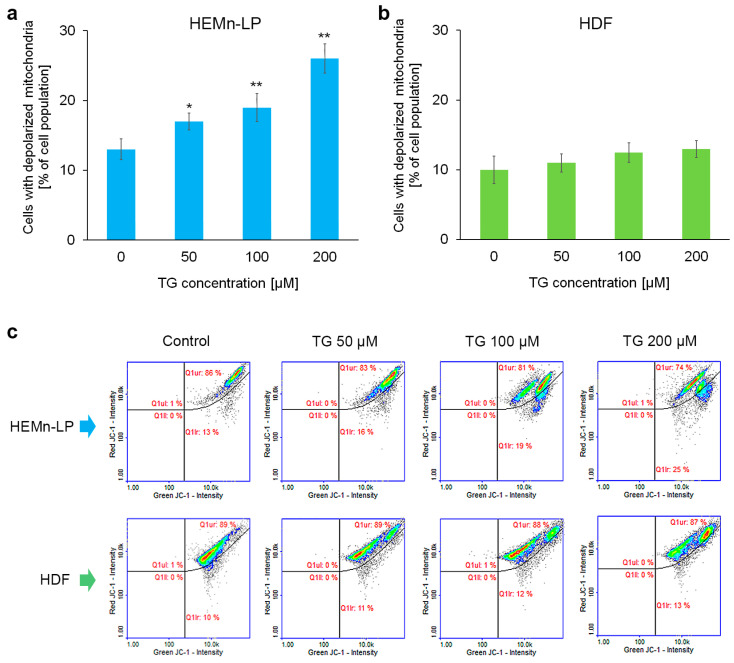
The effect of tigecycline (TG) on mitochondrial inner transmembrane potential in (**a**) human melanocytes, HEMn-LPs, and (**b**) fibroblasts, HDFs. All analyses were performed after a 48 h incubation of the cells with TG at a concentration of 50, 100, or 200 µM. Bar graphs show the mean value ± SD of three independent experiments; * *p* < 0.05, ** *p* < 0.01 vs. control. Representative scatterplots from the analysis (**c**) show the populations of cells with polarized (Q1ur) and depolarized (Q1lr) mitochondria. Colors in scatterplots indicate how many cells are located in a given area of the plot: red / yellow = high density (many cells), blue = low density.

Mitochondrial inner transmembrane potential (ΔΨm) depolarization is an initial and irreversible step towards apoptosis. In the present study, we used image cytometry and the JC-1 dye to assess this parameter in the cells. As shown in Figure 3, tigecycline induced ΔΨm depolarization in melanocytes. This effect was proportional to the drug concentration (Figure 3a). For fibroblasts, no statistically significant change was observed for any of the drug concentrations compared to the untreated control (Figure 3b).

### 2.4. Reduced Thiols Status in Melanocytes and Fibroblasts Treated with Tigecycline

**Figure 4:**
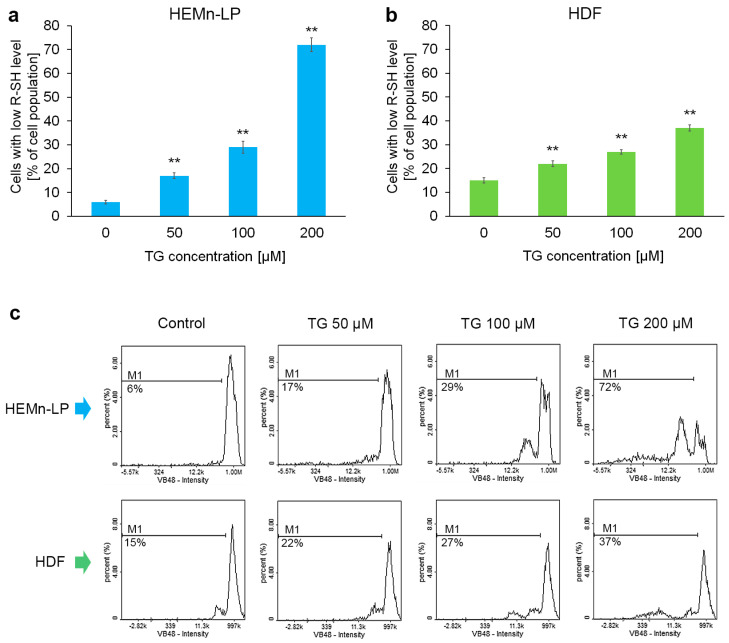
The impact of tigecycline (TG) on intracellular reduced thiols (R-SH) levels of (**a**) human melanocytes, HEMn-LPs, and (**b**) fibroblasts, HDFs. The cells were cultured for 48 h with TG at a concentration of 50, 100, and 200 μM. Bars represent the mean ± SD (standard deviation) of 3 independent experiments, ** *p* < 0.01 vs. control. Representative histograms from the analysis (**c**) show the populations of cells with low levels of reduced thiols (M1).

Epidermal melanocytes are particularly vulnerable to oxidative stress owing to the pro-oxidant state generated during melanin synthesis [23]. The results presented in Figure 1, Figure 2 and Figure 3 indicate that fibroblasts are more resistant to the cytotoxic effect of tigecycline than melanocytes. Taking this into account, we decided to assess the impact of tigecycline on the intracellular reduced thiols (R-SH) level, which reflects cellular redox homeostasis. The results presented in Figure 4 show that incubation with tigecycline led to an increase in oxidative processes in melanocytes and fibroblasts; however, the effect was much stronger in melanocytes. In the HEMn-LP population treated with tigecycline at a concentration of 200 µM, more than 70% of the cells had low levels of reduced thiols (Figure 4a). For human dermal fibroblasts exposed to the same conditions, the percentage was approx. 40% (Figure 4b).

Based on published data, tetracycline antibiotics are known to affect oxido-reductive homeostasis of both normal skin cells and skin-derived tumors. Minocycline reduces the number of cells with low levels of reduced intracellular thiols in melanocytes and melanoma [24,25]. Tigecycline and doxycycline have similar effects on melanotic and amelanotic melanoma cells, causing redox disturbances [26,27]. Research has indicated that a reduction in nuclear glutathione levels can lead to impaired cell proliferation. This suggests that the observed decrease in melanocyte proliferation might be attributed to drug-induced disruptions in redox balance.

### 2.5. Results of EPR Examination of the Influence of Tigecycline on Free Radicals in Melanocytes

**Figure 5:**
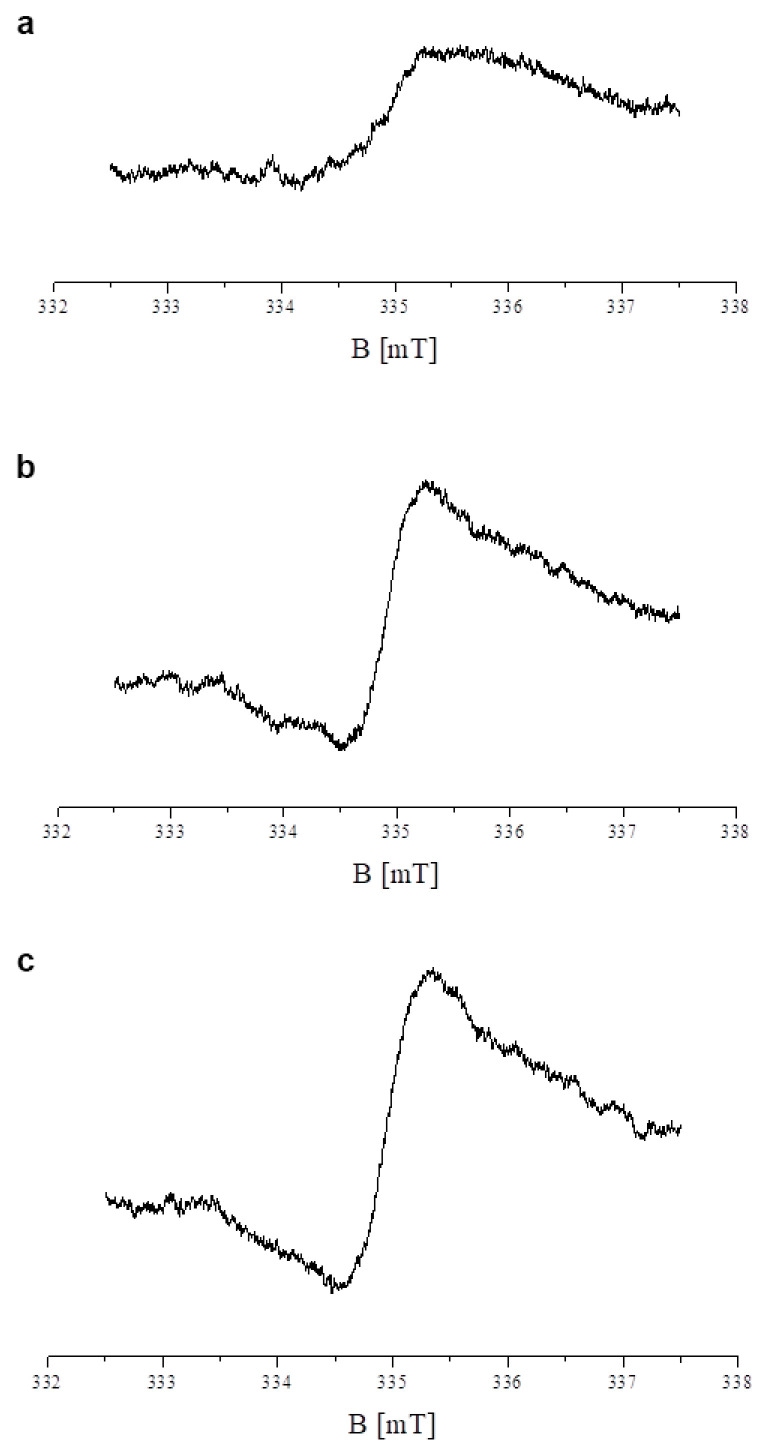
EPR spectra of the control HEMn-LP melanocytes (**a**) and the cells after 48 h incubation with tigecycline at concentrations of 100 µM (**b**) and 200 µM (**c**). The spectra were measured with microwave power of 2.2 mW.

In the current study, we perform EPR analysis to examine the influence of tigecycline on free radical production in melanocytes. EPR spectra were recorded for all HEMn-LP samples tested. Free radicals existed in both the control (untreated) melanocytes and melanocytes incubated with tigecycline for 48 h. The EPR spectrum of the control HEMn-LP cells is shown in Figure 5a. The curve presented in Figure 5a was obtained with a microwave power of 2.2 mW. The EPR spectra of the cells after treatment with tigecycline in concentrations 100 µM and 200 µM, measured with microwave power 2.2 mW, are shown in Figure 5b, and Figure 5c, respectively. The apparent g-factor of these spectra was 2. The parameters of the EPR spectra—integral intensity (I) and linewidth (ΔB_pp_)—for the control cells and the cells treated with tigecycline are in Table 1. The integral intensity (I) of the EPR lines of the cells treated with tigecycline is higher than the integral intensity of the EPR line of the control melanocytes. Higher values of integral intensity were obtained for the cells cultured with tigecycline in a concentration of 200 µM than in the cells cultured with the lower concentration of tigecycline. Tigecycline raised free radical concentration in the examined cells, and this effect increased for the higher drug concentration (Table 1). The narrowing of EPR spectra occurred after treatment of the cells with tigecycline. Linewidths (ΔB_pp_) of the EPR spectra of the cells cultured with tigecycline were 0.71 mT (100 µM tigecycline) and 0.74 mT (200 µM tigecycline), while the linewidth for the spectrum of control HEMn-LP cells had the value of 1.02 mT. Tigecycline changes magnetic interactions in the tested melanocytes.

**Figure 6:**
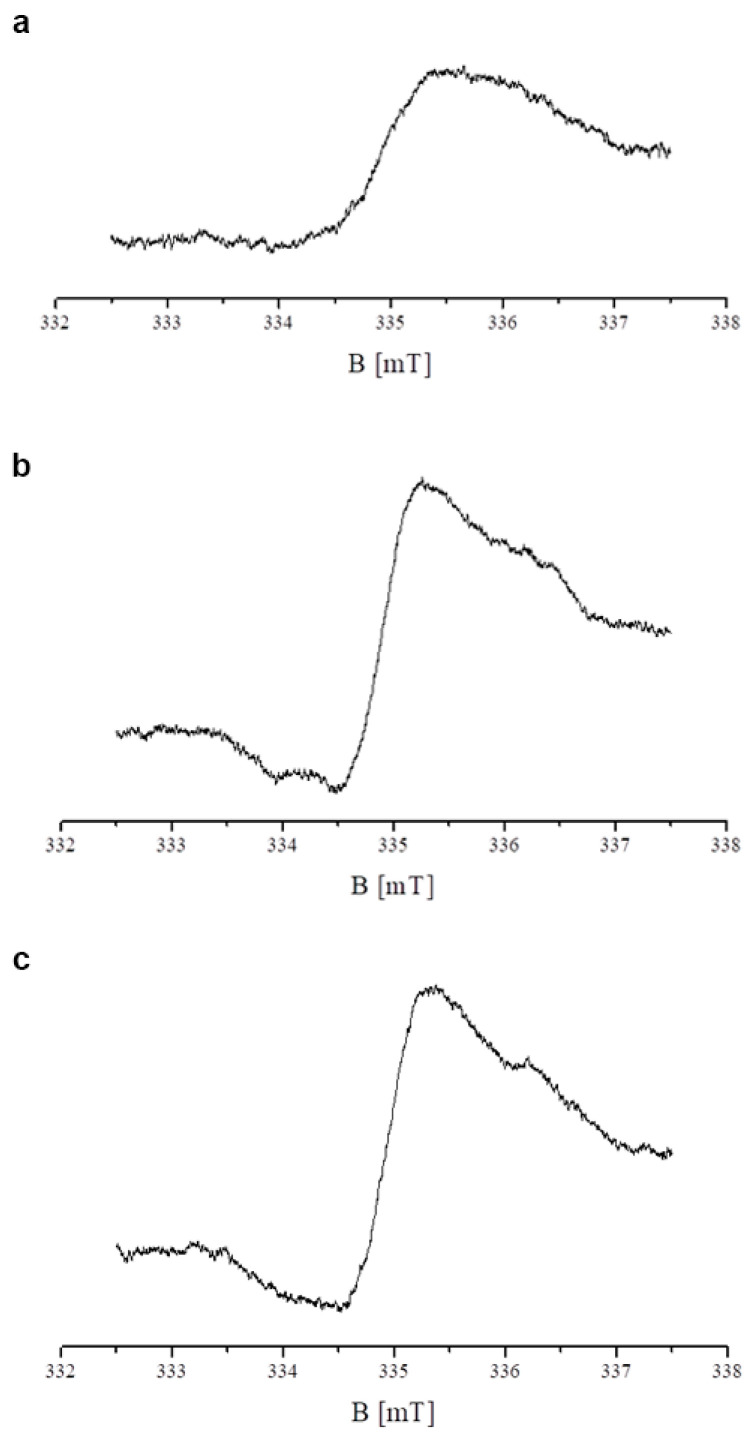
EPR spectra of the control melanocytes (**a**) and melanocytes cultured with tigecycline in concentrations of 100 µM (**b**) and 200 µM (**c**), measured with microwave power of 7 mW.

The spectra are asymmetrical lines (Figure 5a–c). This is probably due to the complex free radicals system in the examined cells. The shape and the parameters of the EPR spectra depended on the microwave power used during the measurement. For example, the EPR spectra of the control and tigecycline-treated melanocytes, measured with the higher microwave power of 7 mW, are shown in Figure 6a–c, respectively. The complex character of the shape of the EPR spectra is visible.

**Figure 7:**
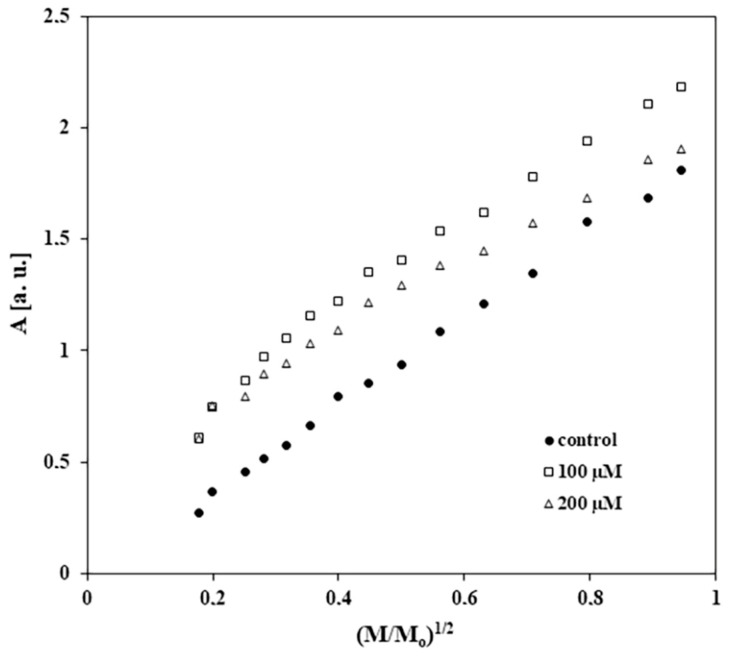
The influence of microwave power on amplitudes (As) of EPR spectra of the control cells and the cells cultured with tigecycline in concentrations of 100 µM and 200 µM. M_o_—the microwave power produced by klystron of the spectrometer (70 mW), M—the microwave power used during the measurement of the line.

**Figure 8:**
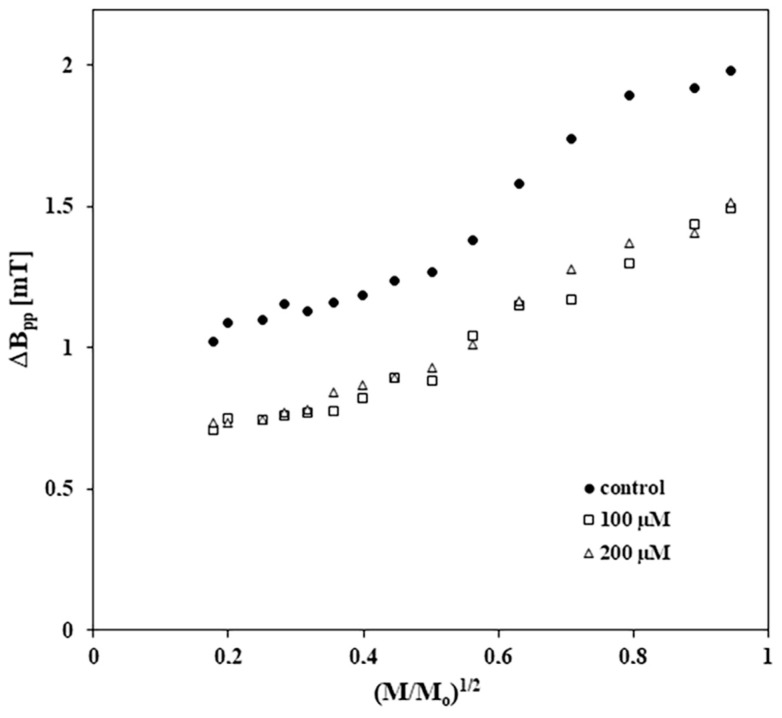
The influence of microwave power on linewidths (ΔB_pp_) of EPR spectra of the control and tigecycline-treated melanocytes. The drug was used in concentrations of 100 µM and 200 µM. M_o_—the microwave power produced by klystron of the spectrometer (70 mW), M—the microwave power used during the measurement of the line.

The changes in the amplitudes (As) of the EPR spectra with increasing of microwave power for the control melanocytes and for melanocytes treated with tigecycline are presented in Figure 7. Figure 8 shows the changes in the linewidths (ΔB_pp_) of the EPR spectra with increasing microwave power for control and tigecycline-treated cells. Amplitudes (As) of all the measured EPR lines increased with increasing microwave power (Figure 7). The microwave saturation of EPR lines was not observed. This indicates the fast spin-lattice relaxation processes in the control cells and the cells treated with tigecycline. In the case of all spectra, the broadening of EPR lines is visible for the higher microwave powers (Figure 8). The EPR lines are homogeneously broadened.

### 2.6. Molecular Docking of Tigecycline to Melanin

**Figure 9:**
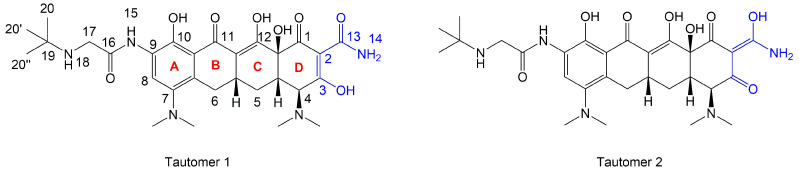
Neutral state of two tautomers of tigecycline.

The next phase of our research was a multistage in silico analysis of the interaction of tigecycline with eumelanin (EM) and pheomelanin (PM). In the first part of the in silico work, we performed simulations, using the Percepta program (version 2024), of the protonation forms in which tigecycline tautomers (Figure 9) may occur in an environment with pH = 1–14. Two forms of protonation state are presented in Table 2.

**Figure 10:**
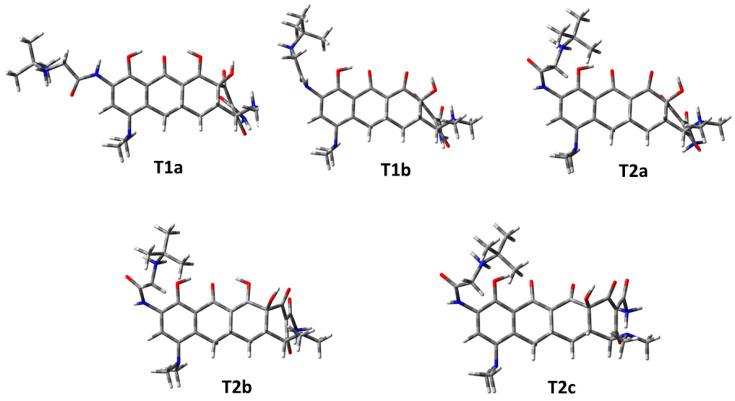
Optimized structures of the ligands used in this study.

The tautomeric structures of tigecycline T1a, T1b, T2a, T2b, and T2c were optimized using the Gaussian program (version 16). The lowest-energy conformers were selected for the next stage of research. The optimized 3D structures are shown in Figure 10.

**Figure 11:**
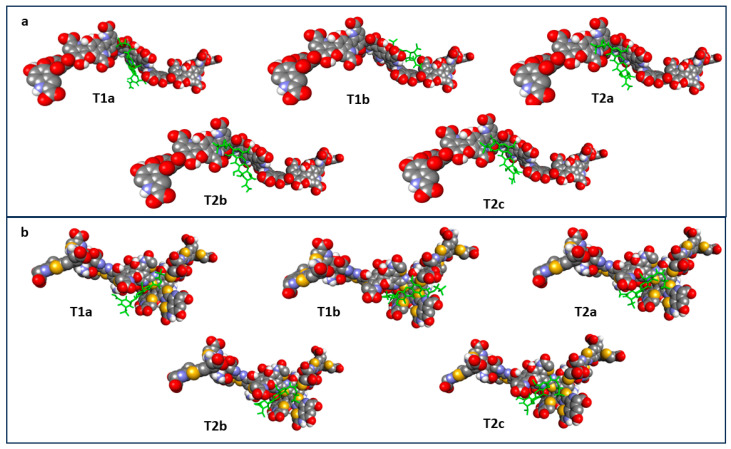
The lowest-energy poses obtained for eumelanin (**a**) and pheomelanin (**b**) complexes with the tested ligands (ligands are colored green).

Molecular docking was performed using AutoDock Vina (version 1.2.7). The entire EM and PM molecule was indicated as the docking target. The docking score values obtained are presented in Table 3, and the lowest-energy poses of ligands in complexes with EM and PM are shown in Figure 11.

**Figure 12:**
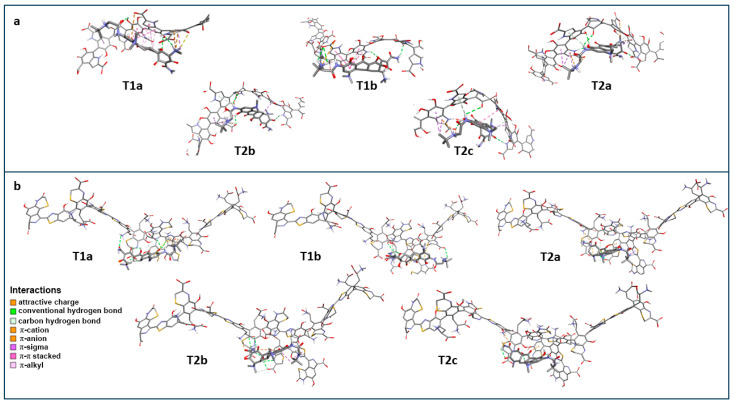
Interactions between eumelanin (**a**) or pheomelanin (**b**) with the tested ligands.

Detailed interactions between the tested ligands and the EM model are presented in Figure 12 and Table 4.

## 3. Discussion

Tigecycline is a valuable antibiotic used to treat various infections, including complicated skin and skin-structure infections. However, like other tetracycline analogues, tigecycline can cause adverse skin reactions, which are important considerations for clinicians. These reactions can range from mild to severe and include hypersensitivity, hyperpigmentation, and rare severe conditions like toxic epidermal necrolysis [28,29,30,31].

Tetracyclines are a class of antibiotics known for their ability to bind to melanin, a biopolymer responsible for pigmentation of skin, hair, and eyes. This binding can lead to drug accumulation in pigmented tissues, potentially causing adverse effects such as phototoxicity [32]. The binding of tetracyclines to melanin is not only a concern for phototoxicity but also for the potential long-term deposition of the drug, which can contribute to pigmentation disorders [24].

In a documented case, a patient developed progressive brown-gray hyperpigmentation of the skin of the trunk and upper arms after 2.5 months of tigecycline treatment. A skin biopsy confirmed the presence of melanin and iron-laden macrophages in the upper dermis. The hyperpigmentation pattern observed was similar to that seen for minocycline, suggesting a common mechanism due to their chemical similarity, as tigecycline is a 9-(*N*,*N*-dimethylglycylamido) derivate of minocycline [33].

To date, there have been no studies confirming the ability of tigecycline to bind to melanin, although this has been confirmed for other tetracyclines—chlortetracycline [32], oxytetracycline [34], and doxycycline [35]. Furthermore, there are no studies on the effects of tigecycline on homeostasis of skin cells, including pigment-producing cells.

Our results demonstrate that tigecycline exerts greater cytotoxicity toward HEMn-LP melanocytes than fibroblasts, suggesting that pigmented cells are particularly vulnerable to its effects. This differential sensitivity may stem from tigecycline’s interaction with melanin, consistent with known pigmentation changes associated with tetracycline antibiotics [28,29,30,31]. The results of the analysis of the cytotoxic effect of tigecycline on normal skin cells using the WST-1 probe showed that the drug reduces the number of metabolically active fibroblasts. In the case of dark-pigmented HEMn-DP melanocytes, it was shown that a statistically significant decrease in survival was observed similarly to our study at a concentration of 50 µM [26]. Studies using tetracycline antibiotics have shown that minocycline, tetracycline, and oxytetracycline at similar concentrations cause a reduction in the number of melanocytes. In addition, these studies were corroborated by photographic documentation suggesting a change in the shape of the cells to spherical, a reduction in their number, and a loss of intercellular contacts [36,37,38].

Studies conducted with tigecycline on melanoma, which are cells of dermal origin, have shown that the drug induces cell cycle arrest at the G0/G1 phase. These results are consistent with those presented here against normal skin cells. The effect is associated with the downregulation of G1/S checkpoint proteins such as CDK2 and Cyclin E, which are crucial for cell cycle progression [24,39]. It is worth noting that another drug from the tetracycline group, doxycycline, is characterized by a proven effect on the inhibition of the activity and expression of metalloproteinases (MMPs) of other skin cells—keratinocytes—which causes cell cycle disruption similarly to tigecycline [40].

In this study we revealed that tigecycline induced mitochondrial depolarization in HEMn-LP melanocytes. Other tetracyclines, minocycline and tygecycline, were shown to reduce mitochondrial membrane potential in melanoma cells and this depolarization was associated with the inhibition of cell proliferation and induction of apoptosis [25,26]. Additionally, doxycycline is known to interfere with mitochondrial translation, leading to disturbed mitochondrial proteostasis and metabolic activity [41].

The enhanced oxidative stress detected in HEMn-LP melanocytes, as indicated by reduced intracellular thiols, reflects disruption of redox homeostasis. Given that melanin synthesis generates a pro-oxidant environment, melanocytes are inherently susceptible to oxidative damage [23]. The redox imbalance may underlie the reduction in melanocyte proliferation and viability observed. Based on published data, tetracycline antibiotics are known to affect oxido-reductive homeostasis of both normal skin cells and skin-derived tumors. Minocycline reduces the number of cells with low levels of reduced intracellular thiols in melanocytes and melanoma [24,25]. Tigecycline and doxycycline have similar effects on melanotic and amelanotic melanoma cells, causing redox disturbances [26,27]. Research has indicated that the reduction in nuclear glutathione levels can lead to impaired cell proliferation. This suggests that the observed decrease in melanocyte proliferation might be attributed to drug-induced disruptions in redox balance.

One of the most remarkable features of melanin as a biopolymer is its persistent EPR signal, resulting from its paramagnetic properties. The free radical content of melanin and its corresponding EPR signal intensity can be easily modified by a number of physicochemical factors, including interaction with drugs [42]. Previous studies indicate the usefulness of EPR spectroscopy to examine modifications in free radical systems of melanin biopolymers caused by interactions with various drugs [43,44,45]. The EPR spectroscopy study presented in this article suggested that tigecycline increases free radical production and alters melanin’s paramagnetic properties. This direct interaction with melanin may facilitate drug accumulation in pigmented tissues and contribute to adverse pigmentation effects. The increase in melanin free radical concentration may be caused by the action of the drug on cells, but this phenomenon requires further studies. It should be noted that the EPR spectra of melanin tend to change after sequestration of xenobiotics. Therefore, a limitation of our study is that the observed changes in the EPR spectra following tigecycline exposure cannot be unequivocally attributed solely to an increase in melanin radical concentration, as melanin–drug interactions may also contribute. Additional experiments using synthetic melanin models are needed to further substantiate these findings.

The key issue in the case of docking low-molecular-weight ligands for specific biological purposes is to determine the form in which the ligand may exist in vivo (ionic, neutral), which in the case of tetracyclines is a complex process. The bioavailability and pharmacology of tetracyclines are strongly dependent on the presence of an essential structural feature, in the form of a ketone-enol system in the A, B, and C rings [46]. This system enables the in vivo formation of ionic and zwitterionic forms, which fundamentally influence the conformation of the molecule and thus influence the biological activity of tetracyclines. Moreover, theoretical calculations indicate that tautomerization may occur in the D ring of tetracyclines within the enol-amide system [47]. Both tautomeric forms are shown in Figure 9. Simulations performed for tautomer 1 showed that tigecycline at physiological pH can exist in 13 different forms (Supplementary File, Figure S1). At pH = 7.4, two forms of protonation state presented in Table 2 may occur in significant amounts (more than 5%). However, among the 17 possible protonation forms of tautomer 2 (Supplementary File, Figure S2), three forms are present in significant amounts at pH = 7.4: T2a, T2b, and T2c (Table 2).

**Figure 13:**
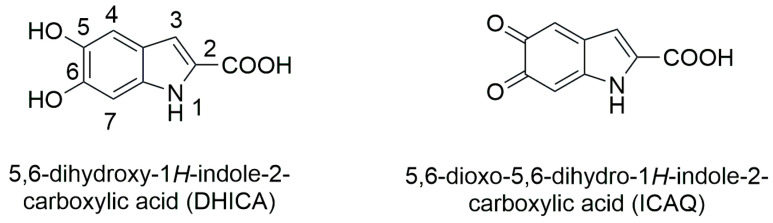
Chemical structures of indole-2-carboxylic acid derivatives used in this work.

The detailed molecular structure of eumelanin is poorly understood, although overwhelming evidence indicates that it corresponds to a highly conjugated aromatic polymer [48]. In the publication of many review papers on eumelanin, several polymer linear or cyclic models created from precursors of 3,4-dihydroxyphenylalanine (DOPA), 5,6-dihydroxyindole (DHI), and 5,6-dioxoindole (IQ) have been proposed. Additionally, two precursors, 5,6-dihydroxyindole-2-carboxylic acid (DHICA) and 5,6-dioxyindole-2-carboxylic acid (ICAQ), also play important roles in melanin biosynthesis [49,50]. TEM imaging of eumelanin-containing DHICA shows aggregation with rod-shaped granular cells [51]. Similarly, SEM analysis of bovine melanosomes showed that they have elongated shapes [52]. It is likely that the carboxylate group in DHICA may be responsible for the twisting of the structure and may lead to weak internal interactions and aggregation. Compared to DHI-based eumelanin, DHICA-eumelanins are characterized by weaker aggregation because of their twisted structure and have stronger redox and photoprotective properties than DHI-eumelanin [53]. DHICA has been reported to have strong hydroxyl radical scavenging activity and higher antioxidant activity compared to DHI-eumelanin [54,55]. Therefore, in this work we focused on building a eumelanin linear model based on DHICA and ICAQ (Figure 13).

DHICA polymerizes mainly through biphenyl-type bonds, which results in the formation of non-planar, linear skeletons that exhibit difficult rotation within the bonds between the benzene rings. The deviation from coplanarity in DHICA oligomers is stabilized by the negative charge of the carboxylate groups, forcing rotation about the bond between the rings. The twisted frameworks thus formed cannot give rise to stacked supramolecular aggregates. The direction of polymerization of DHICA is determined by the presence of a carboxyl group in position 2 of the indole system, which blocks this position and, at the same time, reduces the nucleophilicity of the pyrrole moiety by withdrawing electrons and thus also reduces the reactivity in position 3. Polymerization of DHICA and/or ICAQ units can therefore take place mainly in positions 4,7′, as well as 4,4′ and 7,7′.

**Figure 14:**
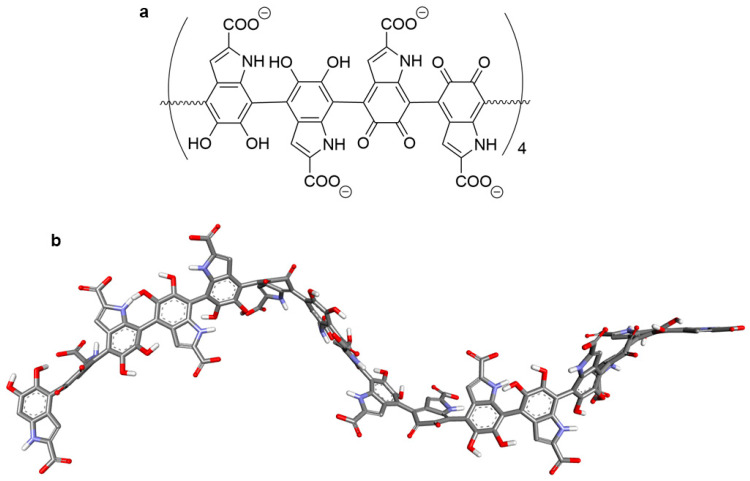
The 2D structure (**a**) and optimized structure (**b**) of eumelanin used in this study.

In our research, we built a model in which the basic monomer consists of two DHICA units and two ICAQ units connected at the 4 and 7′ positions. This monomer was multiplied four times to obtain an oligomer containing sixteen units of 2-carboxyindolic acid derivatives (Figure 14a). The obtained oligomeric structure of eumelanin was optimized using the Gaussian program. The conformer with the lowest energy is shown in Figure 14b.

**Figure 15:**
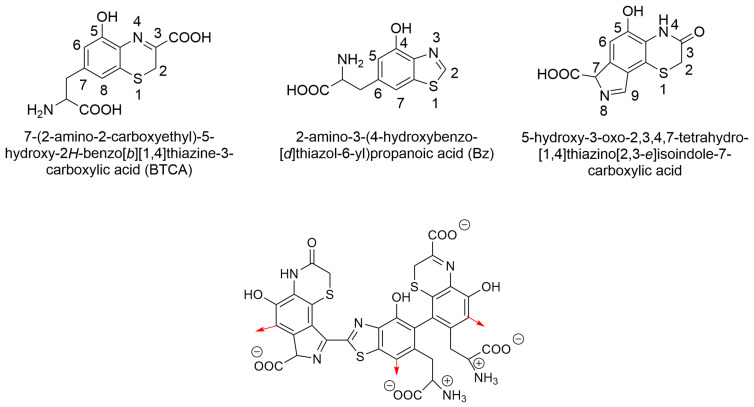
Structures of compounds constituting pheomelanin monomer and model for monomeric pheomelanin structure used in this study. Red arrows indicate possible points for polymer growth.

The structure of PM is more complex than that of EM and is therefore less well understood. Previous studies indicate that it consists of oligomers composed of sulfur-containing units, primarily benzothiazine and benzothiazole with different degrees of oxidation. Benzothiazine derivatives whose presence has been confirmed in PM include 7-(2-amino-2-carboxyethyl)-5-hydroxy-2*H*-benzo[*b*][1,4]thiazine-3-carboxylic acid (BTCA) (DHBTCA) and 2-amino-3-(5-hydroxy-3-oxo-3,4-dihydro-2*H*-benzo[*b*][1,4]thiazin-7-yl) propanoic acid (ODHBT). Benzothiazole, on the other hand, occurs in the form of 2-amino-3-(4-hydroxybenzo[*d*]thiazol-6-yl) propanoic acid (BZ). Furthermore, the presence of benzothiazolylthiazineisoquinoline rings has been proposed, but analogous units with carboxylated isoindole rings could also occur [11,13,14,56,57,58]. This is because this part of the structure could result from direct cyclization of the L-tyrosine side chain of the BTCA or ODHBT, forming isoquinoline or isoindole units. In this work, the model proposed by Solano was used [56], in which the monomeric unit of PM consists of BTCA, BZ, and ODHBT in which the tyrosine side chain is cyclized to the isoindole system (Figure 15).

**Figure 16:**
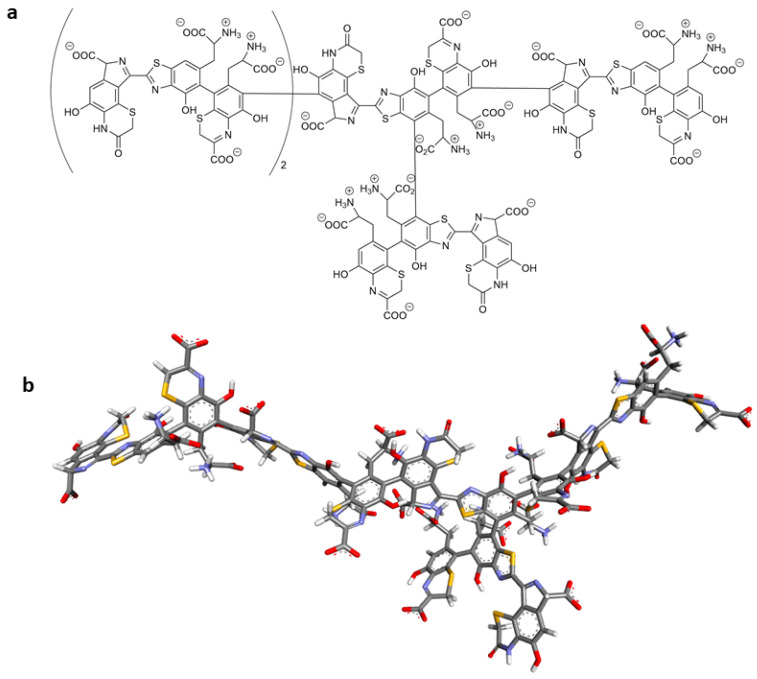
The 2D structure (**a**) and optimized structure (**b**) of pheomelanin used in this study.

In the constructed model, we linearly connected four monomeric units. The C-6 carbon atom of the isoindole-ODHBT molecule and the carbon atom at position 6 of the BTCA contributed to the creation of this linear structure. Additionally, at the C-7 atom of BZ, we introduced a branch in the form of another fifth monomeric unit (Figure 16).

All five ligands were then docked to the constructed EM and PM models. The docking score values obtained are presented in Table 3, and the lowest-energy complexes of ligands with EM and PM are shown in Figure 11.

Overall, the data obtained indicate that tigecycline exhibits a slightly higher binding potential for eumelanin than for pheomelanin. However, the differences in the energy values obtained for the complexes for each ligand and for EM and PM are not large, falling within the range of 0.5–1.0 kcal/mol. As shown in Table 3, the T2c structure exhibits the lowest docking coefficient, that is, the highest potential affinity for EM (−7.5 kcal/mol). The T1b tautomer, in turn, has the lowest potential affinity for EM (−7.0 kcal/mol). The overall stability series of the complexes of the tested ligands for EM is as follows: T1b < T2b < T1a < T2a < T2c, and the difference between the most stable and least stable complexes is not large, 0.5 kcal/mol.

The T2b structure exhibits the highest potential affinity for PM (−6.8 kcal/mol). In turn, the T2a tautomer has the lowest potential affinity for PM (−6.2 kcal/mol). The overall stability series of the tested ligand complexes for PM is as follows: T2a < T1a < T1b = T2c < T2b, and the difference between the most stable and least stable complex is comparable to that obtained for eumelanin and is 0.6 kcal/mol. It is worth noting that the results obtained in silico indicate that the T2c ligand has a high potential ability to bind to both EM and PM.

Detailed interactions between the tested ligands and the EM model are presented in Figure 16 and Table 4. In general, the substituents located on the A and D rings of tigecycline have the greatest impact on the stability of the complexes of ligands with eumelanin. Strong interactions in the form of hydrogen bonds or attractive charge interactions are generated by the ammonium group of the N18 nitrogen atom. The nitrogen N15 atom of the amide group and the phenol group present in the C10 carbon atom also take part in the formation of hydrogen bonds. It is also worth mentioning the weaker, but also significant, interactions of the aromatic system of the A ring with the DHICA benzene ring in the form of π-π stacking interactions. As already mentioned, the substituents of the D-ring of tigecycline are also involved in the formation of strong interactions with eumelanin. The formation of this type of bond involves oxygen atoms located at positions 1 or 3 and the ammonium group at positions 4. It should be noted that interactions of the amide/enol moiety occurring in position 3 were found only in the case of the T1b structure.

In the eumelanin polymeric chain, both the DHICA and ICAQ carboxylate groups, as well as oxygen atoms in positions 5 and 6 and the endocyclic nitrogen atom of the pyrrole ring strongly interact with the ligands. Furthermore, the complexes are stabilized by the weaker interactions generated by both aromatic rings of DHICA (Table 4).

The interactions between the studied ligands and the PM model are presented in Figure 12 and Table 4. Similar to the interaction of tigecycline with eumelanin, substituents located on the A and D rings of tigecycline have the greatest impact on the stability of the ligand–pheomelanin complexes. In the case of substituent interactions occurring on the A-ring of ligands, numerous hydrogen bonding interactions were observed, generated by the hydroxyl group at position 10 and the amino group at position 7. Furthermore, the quaternary ammonium group of the N18 nitrogen atom interacts with the aromatic rings of pheomelanin through π-cation interactions. Substituents on the D ring of tigecycline also participate in the formation of strong interactions with pheomelanin. The hydroxyl group located at position 12a participates in the formation of this type of bond. It should be noted that amide/enol interactions occurring at position 3 were observed for the five ligands, distinguishing pheomelanin from eumelanin, where such interactions were observed only for the T1b structure.

In the case of pheomelanin, as in eumelanin, all carboxylate groups present in BTCA, Bz, and indole-ODHBT molecules interact with ligands through hydrogen bonds. Similar to eumelanin, interactions between aromatic rings and positively or negatively charged substituents of ligands (π-cation and π-anion interactions) also have a similar nature. However, in pheomelanin, a new interaction appears, related to the presence of a sulfur atom and tyrosine chains. Endocyclic sulfur atoms interact with ligands most often through hydrogen bonds, although other interactions, such as π-sulfur and π-cation interactions, also occur. Tyrosine residues, on the other hand, interact with ligands through ammonium groups.

The results obtained from in silico studies indicate a high probability that tigecycline affects melanin. However, differences in the interactions of tigecycline with eumelanin and pheomelanin are evident, as in silico studies indicate a higher potential for tigecycline to bind to eumelanin than pheomelanin.

Our in silico docking analyses support the experimental findings, showing that tigecycline binds melanin mainly through hydrogen bonding and π-π stacking interactions and ionic interactions. The drug’s protonation state and tautomeric form influence binding strength, highlighting the complexity of its interaction with melanin biopolymers. The constructed eumelanin and pheomelanin models provided a biologically relevant scaffold to study these interactions. The involvement of carboxylate and aromatic groups in ligand binding is consistent with the known chemistry of melanin polymers and their capacity to interact with small molecules [48,49,50,51,52,53,54,55].

Overall, these results provide mechanistic insight into tigecycline’s cytotoxicity in pigmented skin cells and its potential to interact with melanin, which may explain clinical observations of tigecycline-induced skin disorders. A limitation of this study is the exclusive use of HEMn-LP melanocytes, which are enriched in pheomelanin. While this model was selected to explore responses under low-pigmentation conditions, future studies should include HEMn-MP and -DP melanocytes to assess the contribution of different melanin types to the observed effects.

## 4. Materials and Methods

### 4.1. Chemicals and Reagents

The source of the tigecycline was Tygacil (50 mg C_29_H_39_N_5_O_8_ × 10 vials) that was purchased from Pfizer (New York, NY, USA). Fibroblast Growth Medium, phosphate-buffered saline (PBS), and the antibiotics amphotericin B and penicillin were obtained from Sigma-Aldrich Inc. (Taufkirchen, Germany). Neomycin sulfate was purchased from Amara (Kraków, Poland). An M-254 growth medium and HMGS-2 supplement dedicated to melanocytes were acquired from Cascade Biologics (Portland, OR, USA). Trypsin/EDTA was obtained from Cytogen (Zgierz, Poland). A8 NC_Slides™, Via-1 Cassettes™ containing the dyes acridine orange and DAPI, as well as staining reagents—Solution 10 (lysis buffer), Solution 11 (stabilization buffer), Solution 12 (500 µg/mL DAPI), Solution 5 (VB-48™/PI/AO), Solution 7 (200 µg/mL JC-1), Solution 8 (1 µg/mL DAPI in PBS)—were acquired from ChemoMetec (Lillerød, Denmark). The remaining chemical reagents were obtained from Sigma-Aldrich (Taufkirchen, Germany) or POCH S.A. (Gliwice, Poland).

### 4.2. Cell Culture

Human dermal fibroblasts (HDFs) purchased from Sigma Aldrich Inc. (St. Louis, MO, USA), and human epidermal melanocytes, lightly pigmented (HEMn-LP), obtained from Cascade Biologis (Portland, OR, USA) provided the material for in vitro studies. Skin cells were cultured in appropriate medium at 37 °C, and 5% humidity. Fibroblasts were cultured in all-in-one Fibroblast Growth Medium. Medium M-254 enriched with growth supplement HMGS-2, and antibiotics: amphotericin B (0.25 mg/mL), neomycin sulfate (10 μg/mL), and penicillin G (10,000 U/mL) provided the culture medium for melanocytes. The cells used in the in vitro studies were from actual passages 5–9 (counted from the initial isolation of the cells by the supplier).

### 4.3. The Assessment of Cell Number and Viability

The image fluorescence cytometer NucleoCounter^®^ NC-3000™ (ChemoMetec, Lillerød, Denmark) considered the assessment of cell number and viability of analyzed skin cells exposed to tigecycline (50, 100, or 200 µM for 48 h). Briefly, the fibroblasts and melanocytes cultures, after detachment by trypsinization process, were centrifuged, and successively resuspended in dedicated growth medium. The cell suspensions were then loaded into Via1-Cassettes™, and stained with two fluorescent dyes—acridine orange, which stains the entire cell population, and DAPI, that has the capacity to penetrate cells with damaged cell membranes. The analysis was carried out in accordance with “Cell Viability and Cell Count Assay” protocol.

### 4.4. The Assessment of Cell Morphology

Photographic documentation of changes in the morphology of the cells studied was kept during the experiment. For this purpose, a light inverted microscope NIKON TS100F (NIKON, Tokyo, Japan) was used.

### 4.5. Cell Cycle Analysis

Analysis of the effect of tigecycline on the cell cycle of melanocytes and fibroblasts was performed using a NucleoCounter^®^ NC-3000™ (ChemoMetec, Lillerød, Denmark) imaging cytometer. Controls and cells incubated with tigecycline (50, 100, or 200 µM for 48 h) were detached, swirled, and counted according to the procedure described in Section 2.3. Cells at 1 × 10^6^ per sample were used in the cell cycle analysis. The cell suspension was swirled and the supernatants were removed before analysis. Cells were resuspended in 250 µL Solution 10 with DAPI dye (10 µg/mL) and incubated for 5 min at 37 °C. Subsequently, 250 µL of Solution 11 was added to each sample and cytometric analysis was performed according to the “Two-step cell cycle analysis” procedure.

### 4.6. Transmembrane Mitochondrial Potential Assessment

The analysis of transmembrane mitochondrial potential in melanocytes and fibroblasts exposed to tigecycline was measured using the fluorescent cytometer NucloCounter^®^ NC-3000™ (ChemoMetec, Lillerød, Denmark). The melanocytes and fibroblasts were treated with tigecycline at a concentration of 50, 100, or 200 µM for 48 h. Subsequently, the cells were detached by trypsinization, and counted in accordance with 2.3 procedure. Then, 1 × 10^6^ cells were stained with JC-1 dye contained in Solution 7. The JC-1 dye differentiates cells into normal and early-apoptotic cells based on the difference in intracellular localization of the dye. In cells with high mitochondrial potential, JC-1 in the form of aggregates accumulates in the mitochondria, emitting red fluorescence. Conversely, in apoptotic cells, the dye emitting green fluorescence is localized as monomers in the cytoplasm. After 10 min of incubation with Solution 7, the cells were washed twice with PBS solution and the obtained cell pellets were resuspended in 0.25 mL Solution 8 and analyzed with an imaging cytometer according to the “Mitochondrial Potential Assay” protocol.

### 4.7. Measurement of Intracellular Reduced Thiols Level

Intracellular reduced thiol status was assessed with fluorescence cytometer with VitaBright 48 dye (ChemoMetec, Lillerød, Denmark). VitaBright-48 staining allows for distinguishing two cell populations—with low and high levels of reduced intracellular thiols, e.g., glutathione (GSH). Melanocytes and fibroblasts after incubation with tigecycline (50, 100, or 200 µM for 48 h) were harvested and counted. Then, 2 × 10^6^ skin cells/mL were suspended in PBS and stained with 10 µL/190 µL Solution 5. After the staining process, intracellular thiols level was measured in accordance with “Vitality Assay” protocol with NC-3000 cytometer.

### 4.8. In Silico Study

The protonation states of the test ligands were generated using the Percepta program [59]. The appropriate three-dimensional structures of the docking ligands were generated using Gaussian 16 (Revision C-01) [60]. The lowest-energy conformations were obtained by performing optimization calculations using the basis (DFT, B3LYP) method and 6–311 + G (d, p). Docking was performed with AutoDock Vina [61,62] supported by PyRx [63]. The volume of the region of interest for EM used for docking was defined as 65 × 25 × 25 Å with a center point at coordinates X = −8.54, Y = 4.21, Z = −5.70 Å. The volume of the region of interest used for docking for PM was defined as 59 × 29 × 25 Å with a center point at coordinates X = 1.91, Y = 0.13, Z = 0.00 Å and included the entire EM and PM molecules. The AutoDock Vina program generates 9 complexes for each ligand. Only the lowest-energy complexes were selected for further analysis. All results obtained are presented in kcal/mol. The molecular coupling results of the title compounds were visualized using the BIOVIA Discovery Studio program (version 2023 SP1) [64].

### 4.9. EPR Examination of Free Radicals in Melanocytes

Free radicals in the tested cells were examined by electron paramagnetic resonance (EPR) spectroscopy at room temperature. The cells were incubated with tigecycline at a concentration of 100 µM or 200 µM for 48 h. Control samples remained unexposed. After incubation, the cells were detached and counted using a NucleoCounter NC-3000 cytometer. Each pellet containing 2 × 10^6^ of cells per sample was rinsed with PBS to remove media residues and unbound material. The cell pellets were transferred to thin-walled glass EPR tubes, and the volume of the cells in these tubes was determined. The empty glass tubes were free of paramagnetic contaminants and showed no EPR signals.

Unpaired electrons of free radicals absorbed microwaves in magnetic field under resonance conditions. The EPR spectra of free radicals were measured as the first-derivative curves with magnetic modulation of 100 kHz. An X-band (9.3 GHz) EPR spectrometer of Radiopan Firm (Poznań, Poland) and the specially programmed numerical data acquisition system of Jagmar Firm (Kraków, Poland) connected to the spectrometer were used to record the EPR curves. The system was equipped with detection and analysis programs. The EPR spectra were obtained with microwave power from 2.2 mW to 70 mW.

For the EPR spectra of the examined cells, analysis of the following parameters was carried out: apparent g-values, amplitude (A), integral intensity (I), linewidth (ΔB_pp_). Spectroscopic programs dedicated to the EPR spectra, LabVIEW 8.5 (National Instruments, Austin, TX, USA), and the Origin (OriginLab, Northampton, MA, USA) programs, were used.

### 4.10. Statistics

Statistical analysis of the results was performed using GraphPad Prism 8.0 (Graph-Pad Software, San Diego, CA, USA). Normality of the data was verified by the Shapiro–Wilk test; homogeneity of variances was checked by the Brown–Forsythe test. Statistical significance of differences between groups was evaluated using one-way ANOVA followed by Dunnett’s test.

## 5. Conclusions

In summary, the results presented in this article indicate that tigecycline affects the homeostasis of skin cells. Normal human dermal fibroblasts were more resistant to tigecycline than HEMn-LP melanocytes. These effects included a reduction in cell survival and proliferation. After tigecycline treatment, melanocytes, unlike fibroblasts, showed DNA fragmentation, decreased mitochondrial inner transmembrane potential, and disturbances in thiol homeostasis, which may be indicative of oxidative stress and apoptosis. The hypothesis needs to be confirmed using specific markers, and future studies could focus on a more detailed investigation of the mechanism of the effect on human epidermal melanocytes. The results of our in cellulo study suggest a potential contribution of melanin to the toxic effects of tigecycline on the skin. Furthermore, EPR analysis showed that tigecycline increased free radical concentrations in HEMn-LP melanocytes in a concentration-dependent manner. In this article, we also presented an in silico study that involved the construction of a eumelanin and pheomelanin model and characterization of the interaction of tigecycline with this pigment. The results demonstrate the ability of tigecycline to form complexes with melanin, which may have clinical implications in terms of drug accumulation in pigmented tissues. We believe that our study will contribute to a better understanding of the mechanism of skin side effects of tigecycline.

## Figures and Tables

**Table 1:** The parameters of the EPR spectra, recorded with microwave power 2.2 mW, for the control HEMn-LP cells and the cells incubated with tigecycline (TG). I—integral intensity, ΔB_pp_—linewidth. Free radical concentration in the cells is proportional to the value of I parameter.

Sample	I [a. u.] ± 0.2	B_pp_ [mT] ± 0.02
Control	8.8	1.02
TG 100 µM	9.5	0.71
TG 200 µM	10.1	0.74

**Table 2:** Structures of ligands used in this study.

Entry	Structure of Ligand	Percentage at pH = 7.4
**1**	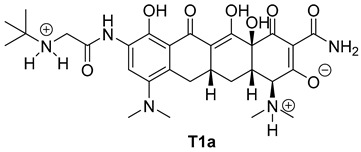	20
**2**	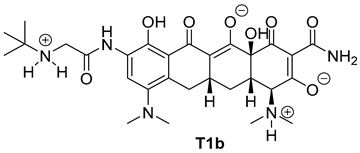	74
**3**	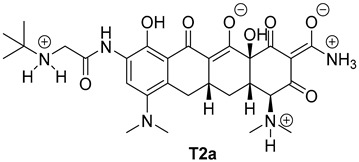	40
**4**	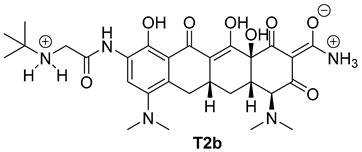	15
**5**	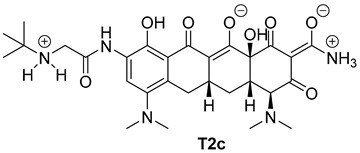	35

**Table 3:** The lowest values of the docking score obtained in the AutoDock Vina program.

Compound	Docking Score [kcal/mol]	
	Eumelanin	Pheomelanin
T1a	−7.2	−6.3
T1b	−7.0	−6.5
T2a	−7.4	−6.2
T2b	−7.1	−6.8
T2c	−7.5	−6.5

**Table 4:** Detailed interactions between eumelanin and pheomelanin and the tested ligands.

**Ligand**		**Eumelanin**	**Interaction**	
**Name**	**Residue**	**Residue**	**Type**	**Distance [Å]**
**T1a**	C16-Carbonyl oxygen	C5-hydroxyl group of DHICA	conventional hydrogen bond	2.37
	C12a hydroxyl group	C6-hydroxyl group of DHICA	conventional hydrogen bond	2.45
	N18 ammonium group	C5-Carbonyl oxygen of ICAQ	conventional hydrogen bond	1.98
	C4-ammonium group	C6-hydroxyl group of DHICA	conventional hydrogen bond	3.07
	C4-ammonium group	C5-hydroxyl group of DHICA	carbon hydrogen bond	3.39
	C4-ammonium group	benzene ring of DHICA	π-cation	3.80
	C4-ammonium group	pirole ring of DHICA	π-cation	4.53
	A-ring	carboxylate group of DHICA	π-anion	4.86
	C20	benzene ring of DHICA	π-sigma	3.90
	Methyl of C4-ammonium group	benzene ring of DHICA	π-sigma	3.94
	A-ring	benzene ring of DHICA	π-π stacked	5.04
	A-ring	benzene ring of DHICA	π-π stacked	5.08
	A-ring	pirole ring of DHICA	π-π stacked	4.33
	Methyl of C7-amine group	pirole ring of DHICA	π-alkyl	4.23
**T1b**	C13 amide group	C6-Carbonyl oxygen of ICAQ	conventional hydrogen bond	2.22
	C1-Carbonyl oxygen	NH of pirole ring of DHICA	conventional hydrogen bond	2.57
	N18 ammonium group	carboxylate group of DHICA	conventional hydrogen bond	2.63
	N18 ammonium group	carboxylate group of DHICA	conventional hydrogen bond	3.05
	N18 ammonium group	carboxylate group of DHICA	attractive charge	4.99
	A-ring	benzene ring of DHICA	π-π stacked	4.35
	A-ring	pirole ring of DHICA	π-π stacked	4.75
	A-ring	benzene ring of DHICA	π-π stacked	4.83
	A-ring	pirole ring of DHICA	π-π stacked	5.80
**T2a**	C4-ammonium group	carboxylate group of DHICA	attractive charge	4.99
	N18 ammonium group	carboxylate group of DHICA	attractive charge	5.16
	C10 hydroxyl group	C5-hydroxyl group of DHICA	conventional hydrogen bond	2.69
	C10 hydroxyl group	NH of pirole ring of DHICA	conventional hydrogen bond	2.01
	N15 amide group	C5-Carbonyl oxygen of ICAQ	conventional hydrogen bond	2.72
	C3-carbonyl oxygen	NH of pirole ring of DHICA	conventional hydrogen bond	2.28
	Methyl of C7-amine group	C6-hydroxyl group of DHICA	carbon hydrogen bond	3.79
	Methyl of C4-ammonium group	carboxylate group of DHICA	carbon hydrogen bond	3.70
	C4-ammonium group	benzene ring of DHICA	π-cation	4.73
	N18 ammonium group	pirole ring of DHICA	π-cation	4.89
	N15 amide group	pirole ring of DHICA	π-donor hydrogen bond	3.21
	C20	pirole ring of DHICA	π-sigma	3.64
	C20	benzene ring of DHICA	π-sigma	3.67
	C20′	pirole ring of DHICA	π-sigma	3.97
	C7	benzene ring of DHICA	π-sigma	3.89
**T2b**	N18 ammonium group	carboxylate group of DHICA	attractive charge	4.97
	N15 amide group	C5-Carbonyl oxygen of ICAQ	conventional hydrogen bond	2.83
	C10 hydroxyl group	carboxylate group of DHICA	conventional hydrogen bond	2.53
	C3-carbonyl oxygen	NH of pirole ring of DHICA	conventional hydrogen bond	2.28
	Methyl of C7-amine group	C6-hydroxyl group of DHICA	carbon hydrogen bond	3.70
	N18 ammonium group	pirole ring of DHICA	π-cation	4.78
	N15 amide group	pirole ring of DHICA	π-donor hydrogen bond	3.23
	C20	pirole ring of DHICA	π-sigma	3.68
	C20	benzene ring of DHICA	π-sigma	3.59
	C20′	pirole ring of DHICA	π-sigma	3.96
	C7	benzene ring of DHICA	π-sigma	3.73
**T2c**	N18 ammonium group	carboxylate group of DHICA	salt bridge;attractive charge	2.64
	C3-carbonyl oxygen	NH of pirole ring of DHICA	conventional hydrogen bond	2.70
	C10 hydroxyl group	carboxylate group of DHICA	conventional hydrogen bond	3.04
	N15 amide group	C5-Carbonyl oxygen of ICAQ	conventional hydrogen bond	2.45
	Methyl of C7-amine group	C6-hydroxyl group of DHICA	carbon hydrogen bond	3.79
	N18 ammonium group	pirole ring of DHICA	π-cation	2.82
	N15 amide group	pirole ring of DHICA	π-donor hydrogen bond	2.48
	N15 amide group	benzene ring of DHICA	π-donor hydrogen bond	3.16
	C20	pirole ring of DHICA	π-sigma	3.55
	C20	benzene ring of DHICA	π-sigma	3.84
	C7	benzene ring of DHICA	π-sigma	3.67
	A-ring	benzene ring of DHICA	π-π stacked	5.41
**Ligand**		**Pheomelanin**	**Interaction**	
**Name**	**Residue**	**Residue**	**Type**	**Distance [Å]**
**T1a**	C13-Carbonyl oxygen	tyrosine ammonium group at C6 of BZ	conventional hydrogen bond	3.05
	C10 hydroxyl group	N3 of Bz	conventional hydrogen bond	2.45
	C10 hydroxyl group	N8 of isoindole-ODHBT	conventional hydrogen bond	2.77
	C12a hydroxyl group	carboxylate group of BTCA	conventional hydrogen bond	2.62
	C7-amine group	C5-hydroxyl group of BTCA	carbon hydrogen bond	3.77
	N18-ammonium group	thiazole ring of Bz	π-cation	3.40
	N18-ammonium group	benzene ring of Bz	π-cation	3.08
	A-ring	S1 of Bz	π-cation	5.54
**T1b**	N18-ammonium group	carboxylate group of BTCA	conventional hydrogen bond	2.86
	N14-amide group	Carboxylate group of isoindole-ODHBT	conventional hydrogen bond	2.53
	C4	C5-hydroxyl group of BTCA	carbon hydrogen bond	3.46
	C4 ammonium group	C5-hydroxyl group of BTCA	carbon hydrogen bond	3.51
	C12 enolate anion	thiazole ring of BTCA	π-anion	4.87
	A-ring	S1 of BTCA	π-sulfur	4.13
	C6	C2 of BTCA	alkyl	5.37
**T2a**	C13 enolate anion	tyrosine ammonium group at C6 of BZ	attractive charge	3.89
	C12 enolate anion	benzene ring of Bz	π-anion	3.97
	C20	thiazole ring of Bz	π-sigma	3.64
	C12a hydroxyl group	Carboxylate group of BTCA	conventional hydrogen bond	3.18
	C12a hydroxyl group	S1 of BTCA	conventional hydrogen bond	2.70
	C4 ammonium group	carboxylate group of BTCA	conventional hydrogen bond	2.71
	Methyl of C7-amine group	C5-hydroxyl group of BTCA	carbon hydrogen bond	3.58
	Methyl of C4-ammonium group	carboxylate group of BTCA	carbon hydrogen bond	2.79
**T2b**	C13 enolate anion	tyrosine ammonium group at C6 of BZ	salt bridge, attractive charge	2.34
	C12a hydroxyl group	S1 of BTCA	conventional hydrogen bond	3.04
	C12a hydroxyl group	carboxylate group of BTCA	conventional hydrogen bond	2.33
	N14 ammonium group	carboxylate group of Bz	conventional hydrogen bond	2.79
	N14 ammonium group	carboxylate group of Bz	conventional hydrogen bond	2.39
	methyl of C4 amine group	carboxylate group of BTCA	carbon hydrogen bond	3.19
	N18 ammonium group	thiazole ring of Bz	π-cation	4.86
	C20	thiazole ring of Bz	π-sigma	3.73
**T2c**	C13 enolate anion	tyrosine ammonium group at C6 of BZ	attractive charge	3.32
	C12a hydroxyl group	carboxylate group of BTCA	conventional hydrogen bond	3.21
	C20	thiazole ring of Bz	π-sigma	3.68

## Data Availability

Data will be made available on request.

## Supplementary Materials

The following supporting information can be downloaded at https://www.mdpi.com/article/10.3390/ijms26188939/s1.
