# Supplementary material for: Effect of Tigecycline on the Homeostasis of Human Epidermal Melanocytes and Fibroblasts
Source: Int J Mol Sci. 2025 Sep 13;26(18):8939. doi: 10.3390/ijms26188939 (PMC12469777; doi:10.3390/ijms26188939)
Supplement: Supplementary file 1 [file ijms-26-08939-s001.zip › ijms-3751930-supplementary.pdf]

Figure S1. Protonation forms of tautomer 1.

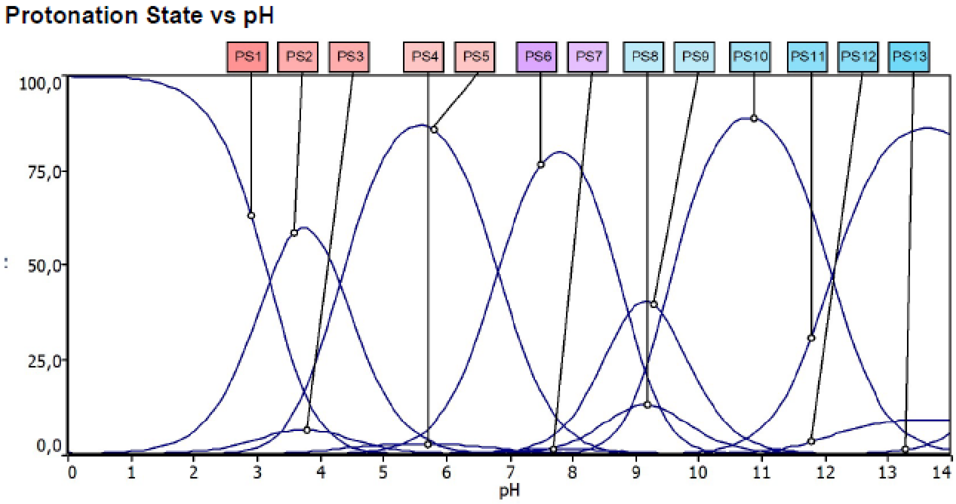

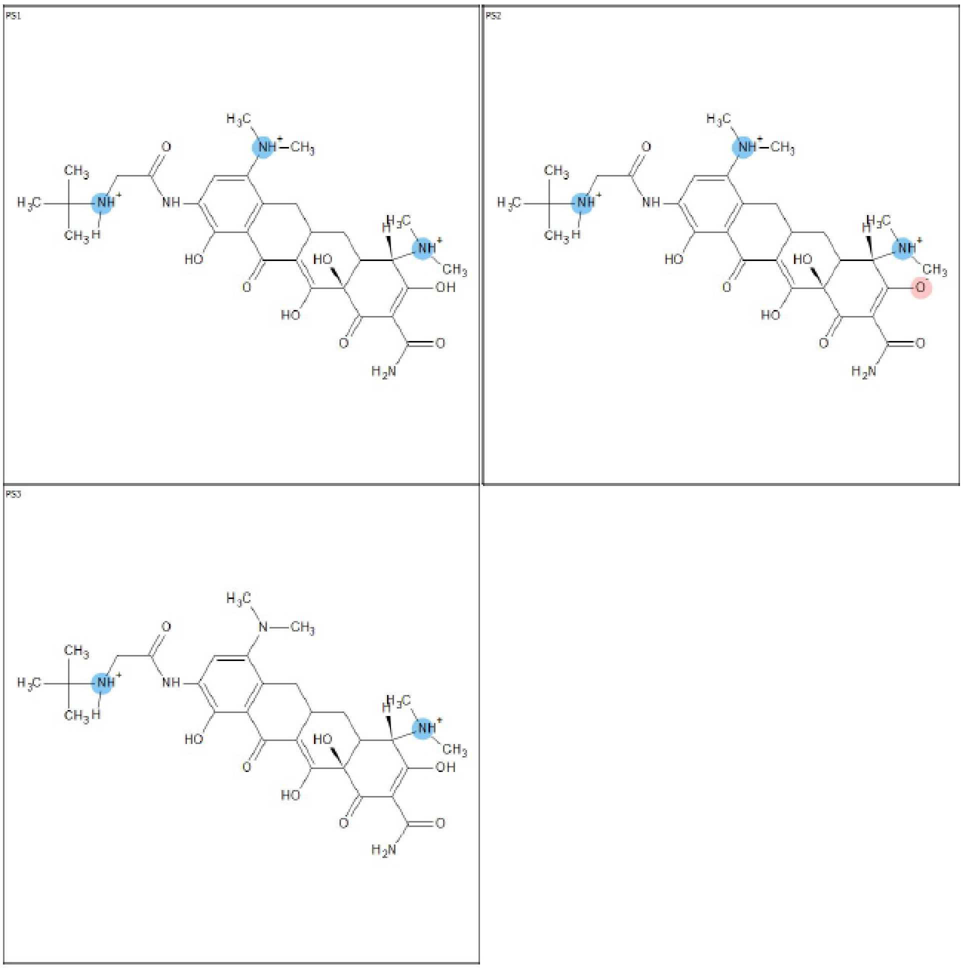

PS4

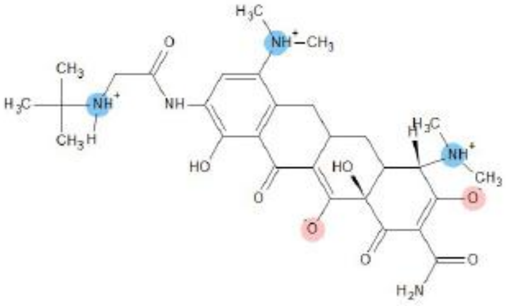

PS5

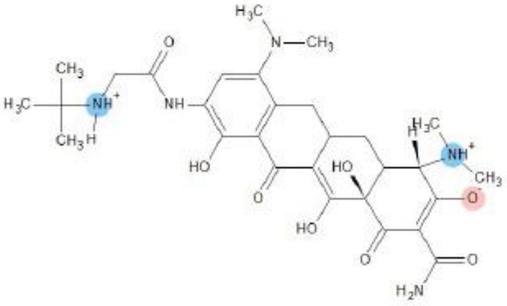

PS6

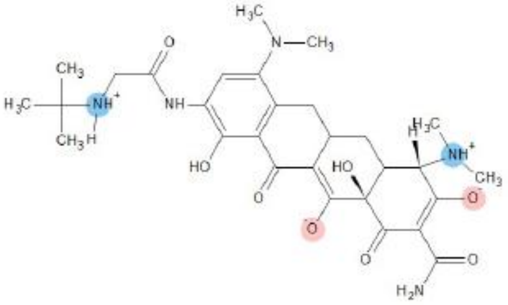

PS7

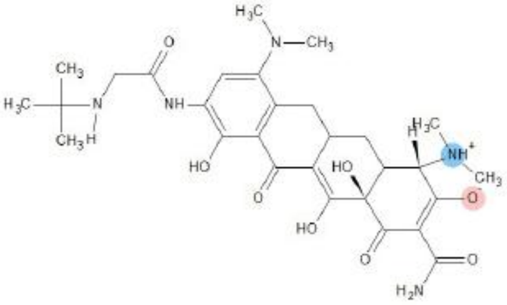

PS8

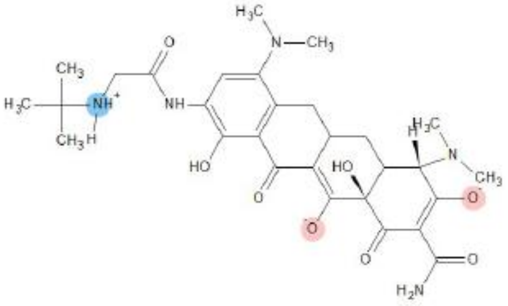

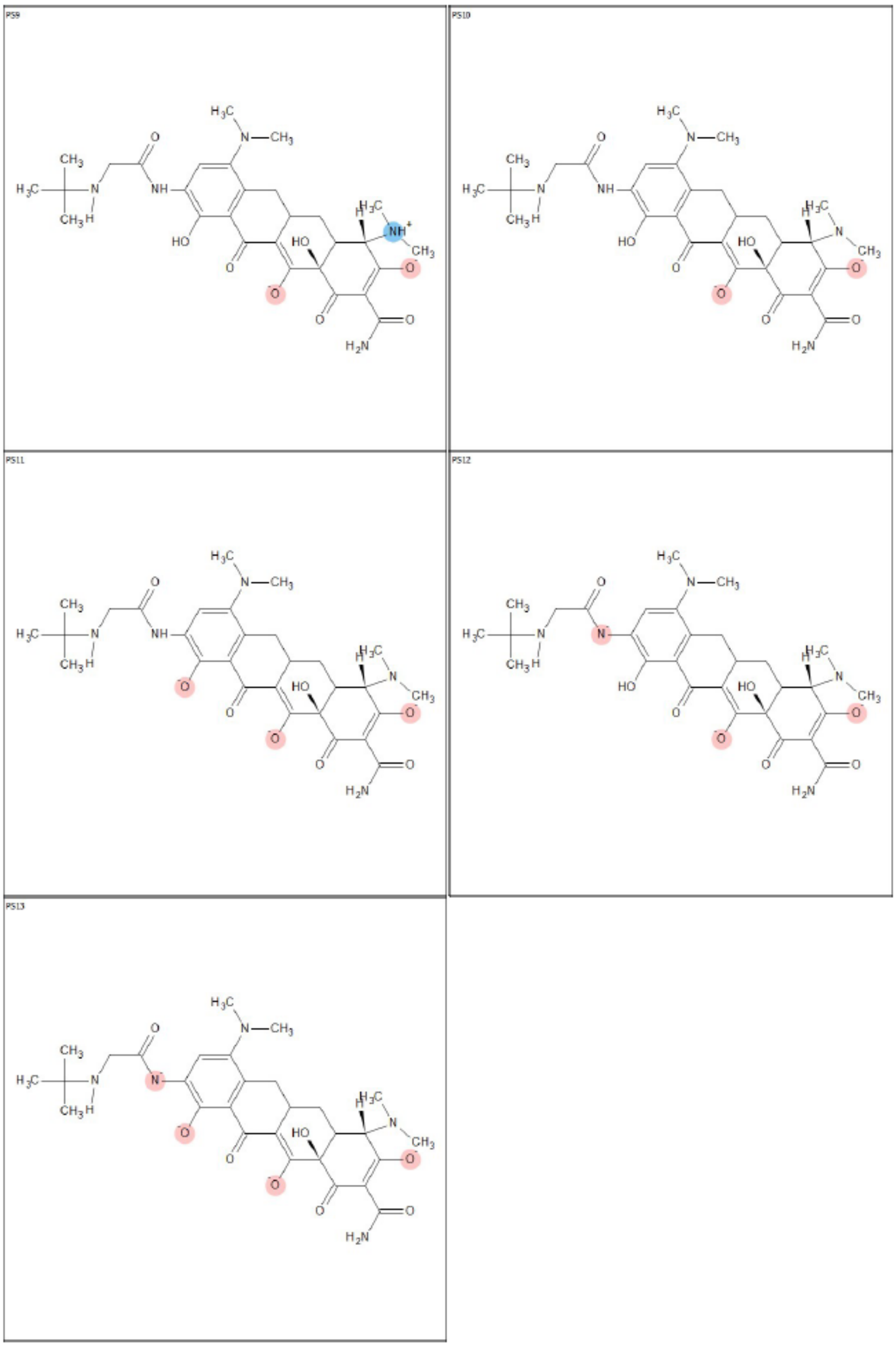

	1,7	2	3	4	4,6	5	6	6,5	7	7,4	8	9	10	11
**PS1**	0,97	0,93	0,57	0,09	0,01	0,00	0,00	0,00	0,00	0,00	0,00	0,00	0,00	0,00
**PS2**	0,03	0,06	0,37	0,57	0,32	0,16	0,02	0,00	0,00	0,00	0,00	0,00	0,00	0,00
**PS3**	0,00	0,01	0,04	0,06	0,03	0,02	0,00	0,00	0,00	0,00	0,00	0,00	0,00	0,00
**PS4**	0,00	0,00	0,00	0,01	0,02	0,02	0,02	0,02	0,01	0,01	0,00	0,00	0,00	0,00

**PS5**	0,00	0,00	0,02	0,27	0,61	0,78	0,83	0,66	0,39	0,20	0,05	0,00	0,00	0,00
**PS6**	0,00	0,00	0,00	0,00	0,00	0,01	0,12	0,31	0,57	0,74	0,78	0,31	0,02	0,00
**PS7**	0,00	0,00	0,00	0,00	0,00	0,00	0,00	0,00	0,01	0,01	0,01	0,00	0,00	0,00
**PS8**	0,00	0,00	0,00	0,00	0,00	0,00	0,00	0,00	0,00	0,01	0,03	0,13	0,06	0,01
**PS9**	0,00	0,00	0,00	0,00	0,00	0,00	0,00	0,00	0,01	0,02	0,10	0,39	0,19	0,02
**PS10**	0,00	0,00	0,00	0,00	0,00	0,00	0,00	0,00	0,00	0,00	0,00	0,15	0,71	0,88
**PS11**	0,00	0,00	0,00	0,00	0,00	0,00	0,00	0,00	0,00	0,00	0,00	0,00	0,01	0,07
**PS12**	0,00	0,00	0,00	0,00	0,00	0,00	0,00	0,00	0,00	0,00	0,00	0,00	0,00	0,01
**PS13**	0,00	0,00	0,00	0,00	0,00	0,00	0,00	0,00	0,00	0,00	0,00	0,00	0,00	0,00

Figure S2. Protonation forms of tautomer 2.

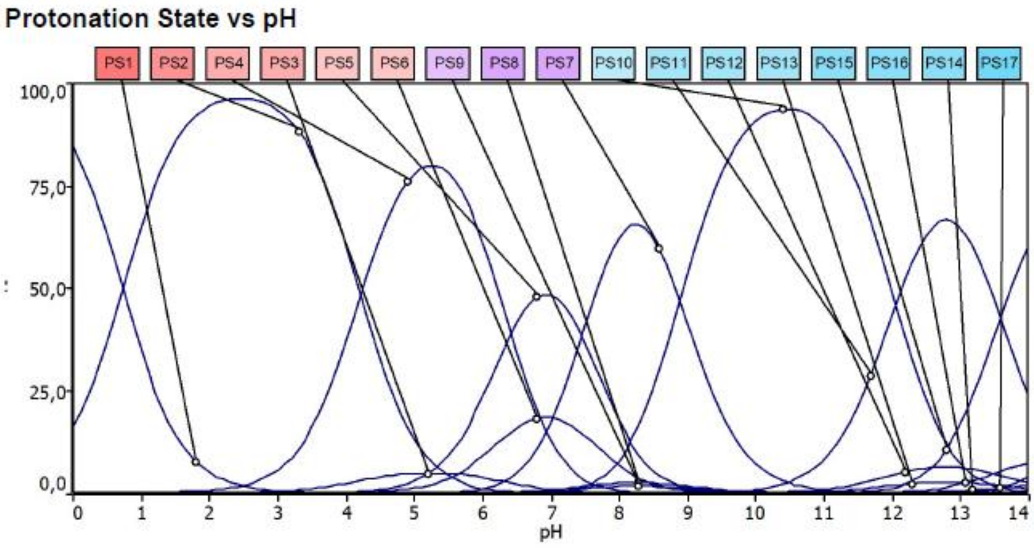

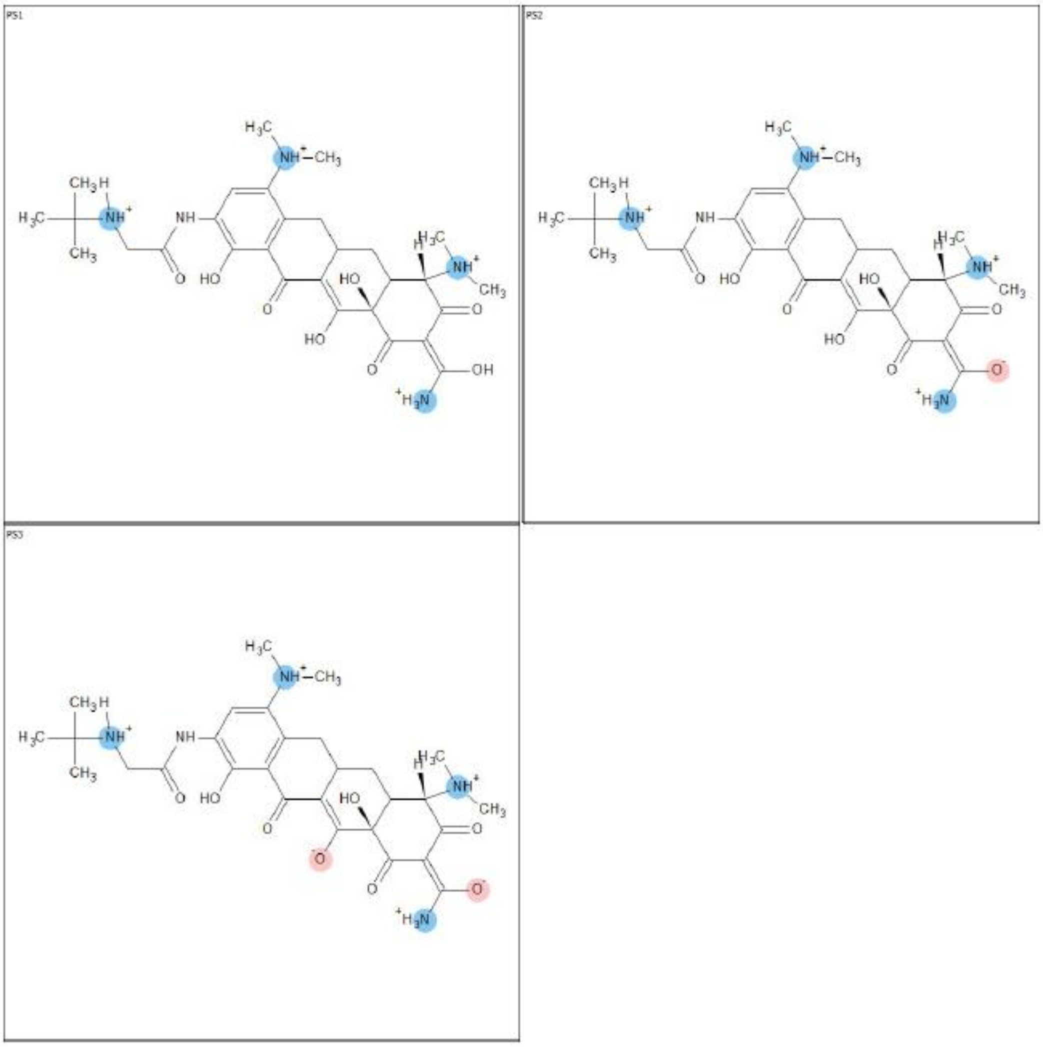

PS4

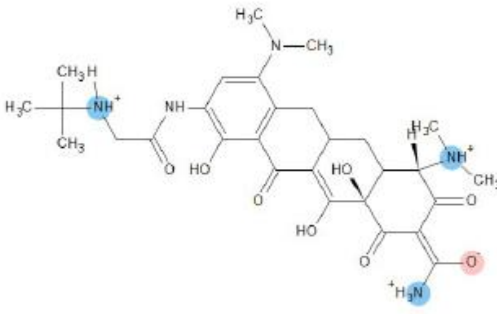

PS5

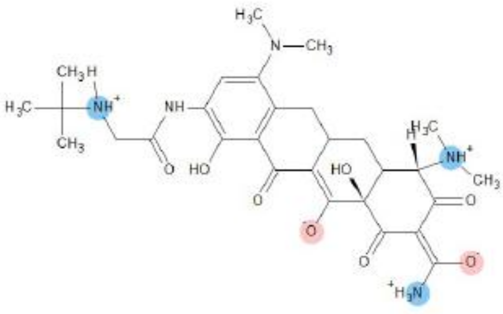

PS6

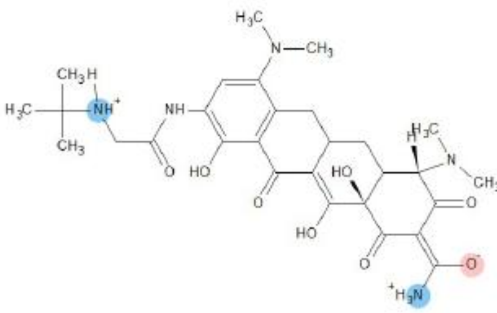

PS7

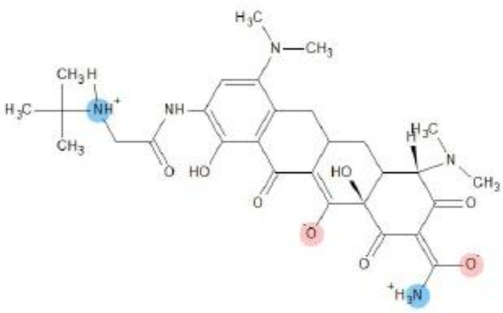

PS8

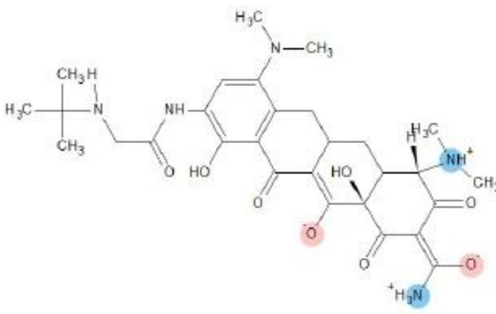

PS9

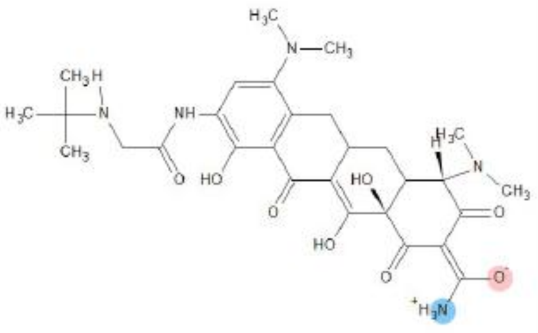

PS10

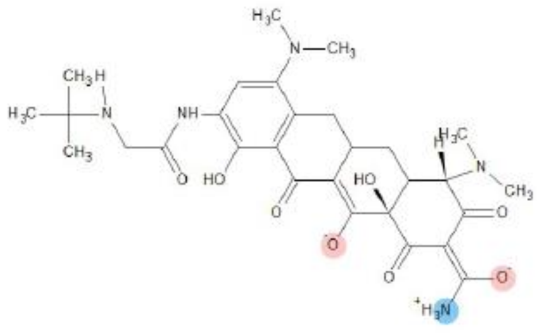

PS11

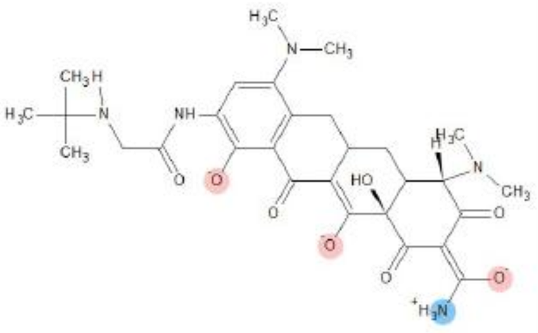

PS12

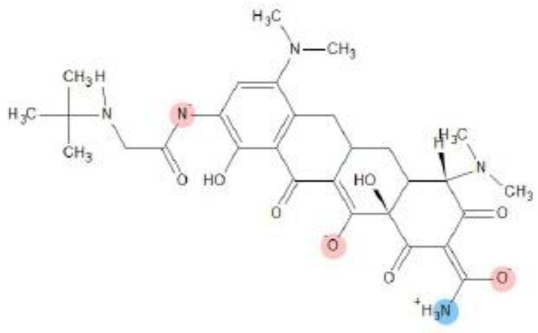

PS13

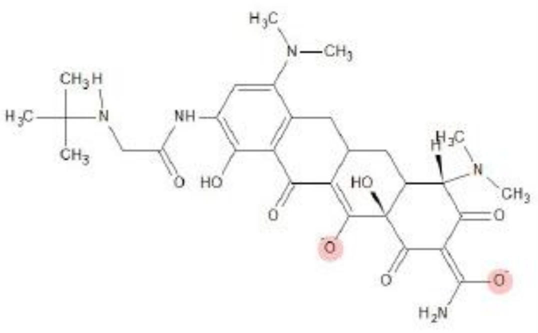

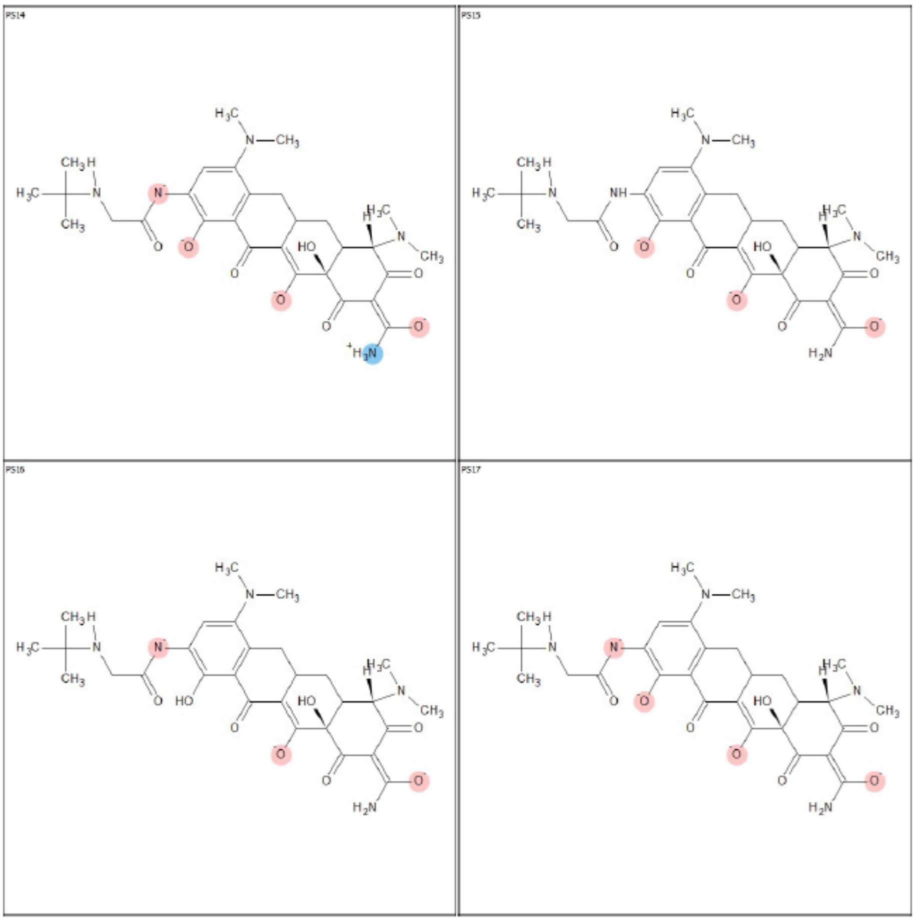

	1,7	2	3	4	4,6	5	6	6,5	7	7,4	8	9	10	11
**PS1**	0,09	0,05	0,00	0,00	0,00	0,00	0,00	0,00	0,00	0,00	0,00	0,00	0,00	0,00
**PS2**	0,90	0,94	0,93	0,61	0,28	0,13	0,01	0,00	0,00	0,00	0,00	0,00	0,00	0,00
**PS3**	0,00	0,00	0,00	0,02	0,04	0,05	0,04	0,02	0,01	0,00	0,00	0,00	0,00	0,00
**PS4**	0,00	0,01	0,06	0,37	0,66	0,78	0,62	0,35	0,13	0,04	0,00	0,00	0,00	0,00
**PS5**	0,00	0,00	0,00	0,00	0,01	0,03	0,23	0,41	0,48	0,40	0,18	0,01	0,00	0,00
**PS6**	0,00	0,00	0,00	0,00	0,00	0,01	0,09	0,16	0,18	0,15	0,07	0,00	0,00	0,00
**PS7**	0,00	0,00	0,00	0,00	0,00	0,00	0,01	0,05	0,17	0,35	0,62	0,42	0,07	0,01
**PS8**	0,00	0,00	0,00	0,00	0,00	0,00	0,00	0,00	0,01	0,01	0,02	0,02	0,00	0,00
**PS9**	0,00	0,00	0,00	0,00	0,00	0,00	0,00	0,00	0,00	0,01	0,02	0,01	0,00	0,00
**PS10**	0,00	0,00	0,00	0,00	0,00	0,00	0,00	0,00	0,00	0,01	0,08	0,53	0,91	0,90
**PS11**	0,00	0,00	0,00	0,00	0,00	0,00	0,00	0,00	0,00	0,00	0,00	0,00	0,01	0,08
**PS12**	0,00	0,00	0,00	0,00	0,00	0,00	0,00	0,00	0,00	0,00	0,00	0,00	0,00	0,01
**PS13**	0,00	0,00	0,00	0,00	0,00	0,00	0,00	0,00	0,00	0,00	0,00	0,00	0,00	0,00

**PS14**	0,00	0,00	0,00	0,00	0,00	0,00	0,00	0,00	0,00	0,00	0,00	0,00	0,00	0,00
**PS15**	0,00	0,00	0,00	0,00	0,00	0,00	0,00	0,00	0,00	0,00	0,00	0,00	0,00	0,00
**PS16**	0,00	0,00	0,00	0,00	0,00	0,00	0,00	0,00	0,00	0,00	0,00	0,00	0,00	0,00
**PS17**	0,00	0,00	0,00	0,00	0,00	0,00	0,00	0,00	0,00	0,00	0,00	0,00	0,00	0,00
